# Structure and Functional Diversity of GCN5-Related *N*-Acetyltransferases (GNAT)

**DOI:** 10.3390/ijms17071018

**Published:** 2016-06-28

**Authors:** Abu Iftiaf Md Salah Ud-Din, Alexandra Tikhomirova, Anna Roujeinikova

**Affiliations:** 1 Infection and Immunity Program, Monash Biomedicine Discovery Institute; Department of Microbiology, Monash University, Clayton, Victoria 3800, Australia; abu.ud-din@monash.edu (A.I.M.S.U.-D.); alexandra.tikhomirova@monash.edu (A.T.); 2 Department of Biochemistry and Molecular Biology, Monash University, Clayton, Victoria 3800, Australia

**Keywords:** GNAT, acetyltransferase, crystal structure, reaction mechanism, enzyme inhibitor, catalytic residues

## Abstract

General control non-repressible 5 (GCN5)-related *N*-acetyltransferases (GNAT) catalyze the transfer of an acyl moiety from acyl coenzyme A (acyl-CoA) to a diverse group of substrates and are widely distributed in all domains of life. This review of the currently available data acquired on GNAT enzymes by a combination of structural, mutagenesis and kinetic methods summarizes the key similarities and differences between several distinctly different families within the GNAT superfamily, with an emphasis on the mechanistic insights obtained from the analysis of the complexes with substrates or inhibitors. It discusses the structural basis for the common acetyltransferase mechanism, outlines the factors important for the substrate recognition, and describes the mechanism of action of inhibitors of these enzymes. It is anticipated that understanding of the structural basis behind the reaction and substrate specificity of the enzymes from this superfamily can be exploited in the development of novel therapeutics to treat human diseases and combat emerging multidrug-resistant microbial infections.

## 1. Introduction

The acylation reactions, catalyzed by diverse groups of enzymes, play an important role in numerous biological processes. The focus of this review is the structure/activity relationships in the superfamily of general control non-repressible 5 (GCN5)-related *N*-acetyltransferases (GNAT, the term introduced into classification by Neuwald and Landsman in 1997 [1]), with an emphasis on the mechanistic insights obtained from the analysis of the GNAT complexes with substrates or inhibitors. GNAT enzymes catalyze the transfer of an acyl group from acyl coenzyme A (acyl-CoA) to an amino group of a wide range of substrates. Although most GNAT enzymes use acetyl-CoA (AcCoA), there are GNAT families that prefer different acyl donors such as myristoyl CoA or succinyl CoA [2,3,4]. The first two three-dimensional structures of representatives of this superfamily, reported in 1998, were those of aminoglycoside *N*-acetyltransferase from multidrug-resistant *Serratia marcescens*, determined by Wolf and co-workers [5], and histone acetyltransferase 1 (HAT1) from *Saccharomyces cerevisiae*, solved by Dutnall [6].

Over the recent decades, more than 309,000 members of the GNAT superfamily have been identified in all domains of life. There are at least 272,000 GNAT proteins in bacteria (of which 118,000 are found in Proteobacteria), 31,000 proteins in eukaryotes and 6500 proteins in archaea [7]. These enzymes are involved in diverse cellular processes, including stress regulation, transcription control, maintenance of a reducing state of the cytosol, protection of cellular contents against oxidants, development of antibiotic resistance and detoxification of thio-reactive compounds [8,9,10]. GNAT enzymes also have anabolic and catabolic functions in both prokaryotes and eukaryotes [2,8]. For example, glucosamine-6-phosphate *N*-acetyltransferase-1 of *S. cerevisiae* is involved in the biosynthesis of a metabolite, uridine diphosphate (UDP)-*N*-acetylglucosamine [11], whereas spermidine/spermine-*N*^1^-acetyltransferase plays an important role in polyamine catabolism [12].

## 2. Overall Architecture of General Control Non-Repressible 5 (GCN5)-Related *N*-Acetyltransferase (GNAT) Superfamily Enzymes

Structural information is currently available for members of 17 distinct families within the GNAT superfamily. Their respective names, Enzyme Commission (EC) classification, source organisms, substrates and the Protein Data Bank (PDB) codes are listed in Table 1.

**Figure 1:**
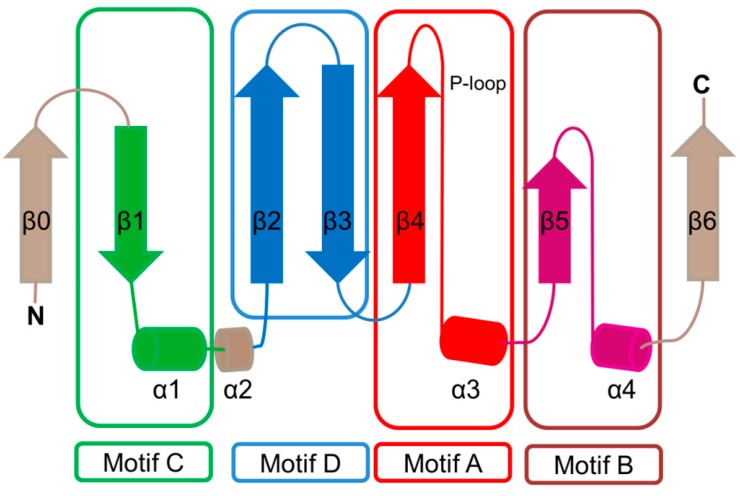
Topology of the general control non-repressible 5 (GCN5)-related *N*-acetyltransferase (GNAT) enzymes. The secondary structure elements encompassing the conserved sequence motifs C (β1–α1), D (β2–β3), A (β4–α3) and B (β5–α4) are colored green, blue, red and magenta, respectively. The location of the conserved “P-loop” (β4–α3) is shown. The least conserved secondary structure elements (strands β0 and β6 and helix α2), that are absent in some GNAT proteins, are colored wheat.

The characteristic conserved core fold of the GNAT superfamily proteins consists of six to seven β-strands and four α-helices, which are connected in order β0-β1-α1-α2-β2-β3-β4-α3-β5-α4-β6 and arranged in the topology shown in Figure 1. GNAT enzymes show moderate pairwise sequence identity (3%–23%) [10]. They vary significantly at the C-terminus while the N-terminus is relatively conserved. Their amino acid sequences harbor four conserved motifs (A–D), which are arranged in order C-D-A-B [2]. Motifs C and D play an important role in maintaining the protein’s stability, while motifs A and B contain residues involved in acyl-CoA and acceptor substrate binding, respectively. Generally, the N-terminal β1 and β2 strands are connected by a loop incorporating two helices (α1 and α2). However, the length, number and position of α-helices in this loop show significant variations across different GNAT enzymes. For example, the second helix (α2) is missing in the structure of yeast HAT1 [6], while aminoglycoside 6′-*N*-acetyltransferase from *Enterococcus faecium* has an additional α-helix between the strands β1 and β2 [13]. The significant variation in this region of the GNAT enzymes allows formation of different acceptor substrate binding sites and thus reflects variation in their substrate specificity. The β4 strand of motif A is parallel to the short β5 strand of motif B; these two strands create a V-shaped opening in the center of the catalytic domain of the protein to accommodate acyl-CoA [2]. Motif B residues are not well conserved across the entire GNAT superfamily. However, within individual families, the enzymes have many common residues within this region. Together with loop β1β2, the α4 helix of motif B and strand β6 at the C-terminal end form the binding site for the acceptor substrate. The structural variations in this region allow different GNAT proteins to recognize a diverse group of acceptor substrates. Most GNAT enzymes have a β-bulge at the center of strand β4 next to the end of the short parallel β5 strand. The β-bulge generates an oxyanion hole that contributes to the stabilization of the tetrahedral reaction intermediate [3,14]. Another distinctive conserved feature is the pyrophosphate binding site in the loop N-terminal to the α3 helix of motif A. Strand β4, helix α3 and strand β5 form a βαβ motif similar to that of the nucleotide-binding Rossman fold [15]. The signature motif at the pyrophosphate binding site, referred to as the “P-loop”, is made up of six amino acids, the amides of which form hydrogen bonds with the phosphate oxygen atoms of acyl-CoA. The consensus “P-loop” sequence in GNAT enzymes is Gln/Arg-x-x-Gly-x-Gly/Ala, where x is any amino acid [5,10].

### 2.1. Aminoglycoside N-Acetyltransferases Family (AAC, EC 2.3.1.81)

**Figure 2:**
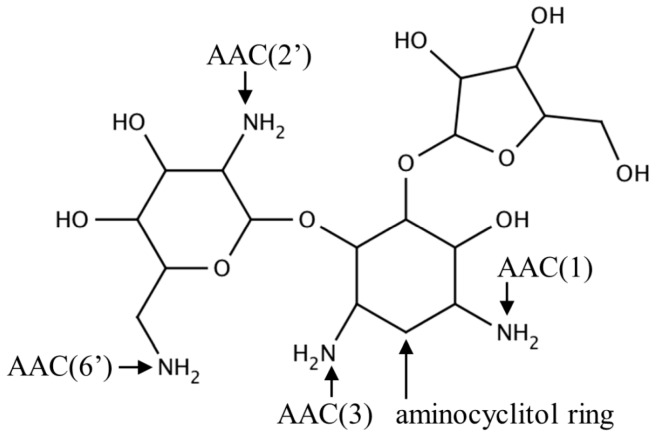
Chemical structure of an aminoglycoside antibiotic (ribostamycin) showing the central aminocyclitol ring and acetyl group modification sites (1, 2′, 3 and 6′).

Aminoglycoside *N*-acetyltransferases (AACs) found in bacteria catalyze regioselective transfer of the acetyl group to one of the four amino groups present on the aminocyclitol ring of a wide variety of aminoglycoside antibiotics (Figure 2), including apramycin, gentamicin, kanamycin, neomycin and tobramycin. Bacteria that possess or acquire the gene encoding AAC show resistance to aminoglycosides because acetylated aminoglycosides have a lower affinity for the tRNA binding site on the bacterial 30S ribosomal subunit [16]. AACs are classified into four major classes based on the site of acetylation: AAC(1), AAC(2′), AAC(3) and AAC(6′) [17]. Recently, a novel type of aminoglycoside modifying enzyme, named enhanced intracellular survival (Eis) protein, was identified in *Mycobacterium* sp. Eis acetylates multiple amino groups of aminoglycosides and thus confers resistance to a wide range of aminoglycoside antibiotics [18,19,20]. Structural information is available on seven different aminoglycoside-modifying enzyme subfamilies: AAC(3)-Ia, AAC(2′)-Ic, AAC(6′)-Ib, AAC(6′)-Ie, AAC(6′)-Ii, AAC(6′)-Iy and Eis [5,10,19,21,22,23,24,25,26], with a representative from each subfamily described below.

**Figure 3:**
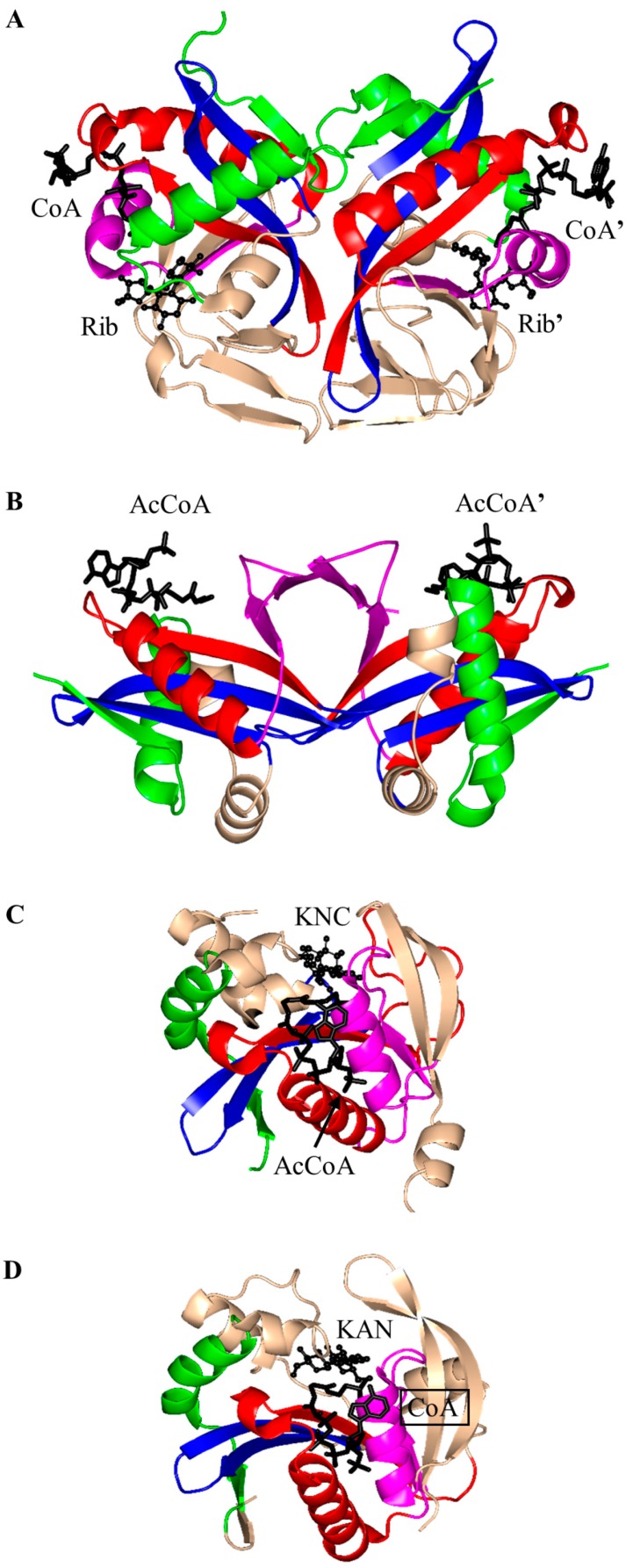

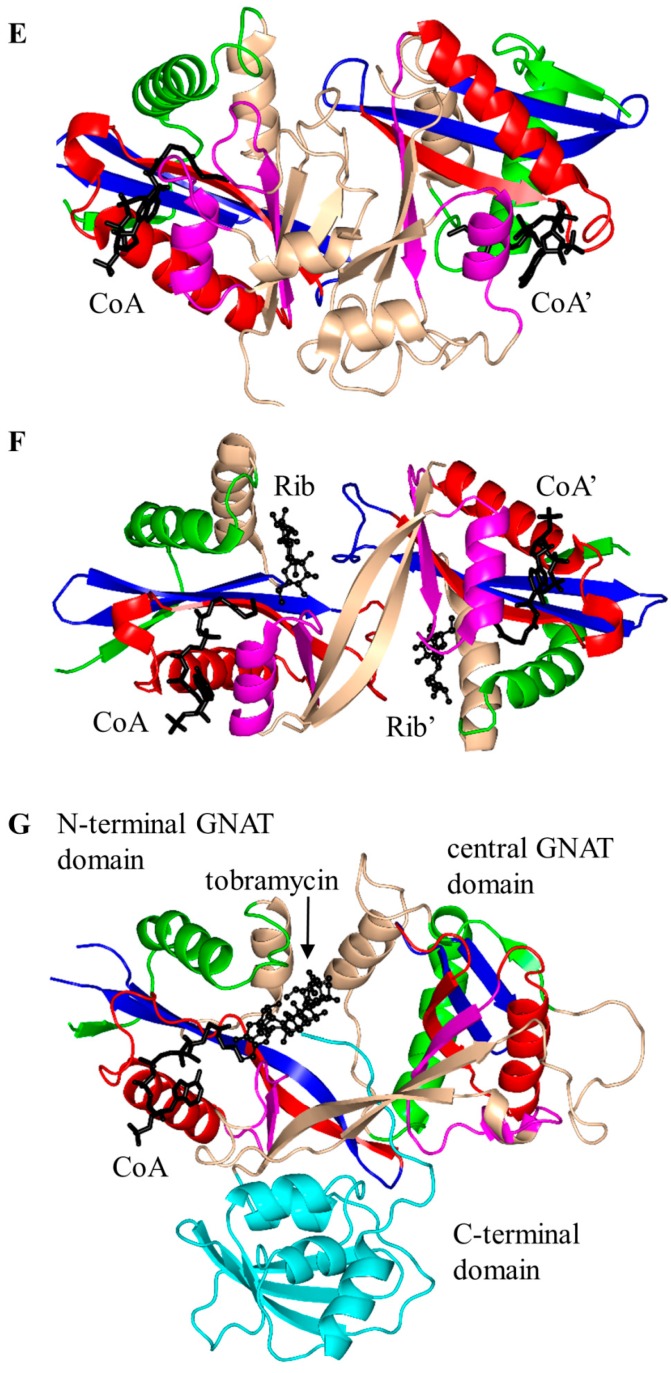
Cartoon representation of the structures of aminoglycoside *N*-acetyltransferases. (**A**) Aminoglycoside 2′-*N*-acetyltransferase-Ic from *Mycobacterium tuberculosis* in complex with CoA and ribostamycin (Rib) (PDB ID: 1M4G [23]); (**B**) aminoglycoside 3-*N*-acetyltransferase-Ia from *Serratia marcescens* in complex with AcCoA (PDB ID: 1BO4 [5]); (**C**) aminoglycoside 6′-*N*-acetyltransferase-Ib from *Escherichia coli* in complex with AcCoA and kanamycin C (KNC) (PDB ID: 1V0C [25]); (**D**) aminoglycoside 6′-*N*-acetyltransferase-Ie from *Staphylococcus warneri* complex with a sulfinic acid form of coenzyme A (CoA) and kanamycin A (KAN) (PDB ID: 4QC6 [24]); (**E**) aminoglycoside 6′-*N*-acetyltransferase-Ii from *Enterococcus faecium* in complex with CoA (PDB ID: 1N71 [34]); (**F**) *Salmonella enterica* aminoglycoside 6′-*N*-acetyltransferase-Iy in complex with CoA and ribostamycin (Rib) (PDB ID: 1S3Z [39]); and (**G**) *M. tuberculosis* enhanced intracellular survival (Eis) in complex with CoA and tobramycin (PDB ID: 4JD6 [19]). The conserved and non-conserved motifs are colored as in Figure 1 (motif C—green, motif D—blue, motif A—red, motif B—magenta, non-conserved N-terminal and C-terminal regions—wheat). The C-terminal animal sterol carrier domain of Eis is colored cyan. The AcCoA/CoA cofactor is drawn as black sticks, whereas the substrates (tobramycin and kanamycin) are shown in black using ball-and-stick representation.

Aminoglycoside 2-*N*-acetyltransferase-Ic of *Mycobacterium tuberculosis* (MtAAC(2′)-Ic) can perform both *N*- and *O*-acetylation of the 2′ amino group of a wide range of aminoglycoside antibiotics [22]. MtAAC(2′)-Ic is a 20.0 kDa protein encoded by the chromosomal *Rv0262c* gene [23]. It is a dimer in the crystal (Figure 3A). Structural analysis of the MtAAC(2′)-Ic ternary complexes with CoA and aminoglycosides revealed that MtAAC(2′)-Ic has a β-bulge in the β4 strand (residues G83 and V84) and a V-shaped cleft between the β4 and β5 strands, that serves as the AcCoA binding site. MtAAC(2′)-Ic has an atypical “P-loop”, the sequence of which (G92-Q93-R94-L95-V96) does not match the consensus found in other GNAT proteins. The “P-loop” interacts with the pyrophosphate arm of CoA via both direct and water-mediated hydrogen bonds [23]. The backbone amide group of V84 forms a hydrogen bond with the carbonyl oxygen of AcCoA and is thought to stabilize the tetrahedral intermediate formed during the acetyl transfer reaction [23]. The hydrogen bond between the backbone amide group of G83 and the 3 amino group of the substrate is important for proper positioning of the acceptor substrate for the direct nucleophilic attack. The hydroxyl group of Y126 is ~3.6 Å away from the sulfur moiety of CoA and could serve as the general acid during catalysis, while the E82 or W181 were suggested to act as the remote general base via well-ordered water molecules [23].

Structural analysis of the plasmid-encoded aminoglycoside 3-*N*-acetyltransferase of *S. marcescens* (SmAAC(3), 168 aa) in complex with CoA revealed that SmAAC(3) forms a dimer in the crystal [5]. SmAAC(3) has a β-bulge in the β4 strand (residue Y109 and D110) and a conserved “P-loop” R118-R119-Q120-G121-I122-A123 that interacts with the diphosphate moiety of CoA. The strands β4 and β5 are splayed apart to form the CoA binding site (Figure 3B). It was shown that a homolog of SmAAC(3), gentamicin 3-*N*-acetyltransferase from *Pseudomonas aeruginosa*, follows a direct acetyl transfer mechanism [27]. Kinetic analysis of gentamicin 3-*N*-acetyltransferase showed that a bisubstrate analog, produced by covalent linking of the reaction product (3-*N*-chloroacetylgentamicin) to CoA, inhibits this enzyme in vitro with high specificity [27].

The 196-residue *Escherichia coli* AAC(6′)-Ib (EcAAC(6′)-Ib) is a chromosome-encoded aminoglycoside-modifying enzyme that confers bacterial resistance to the antibiotics amikacin, kanamycin and tobramycin [25,26,28]. The AAC(6′)-Ib_11_ of *Salmonella enterica* (SeAAC(6′)-Ib_11_), a close homolog of EcAAC(6′)-Ib, confers resistance to a broader range of aminoglycosides that include amikacin and gentamicin [29]. EcAAC(6′)-Ib is a monomer in solution [25], while SeAAC(6′)-Ib_11_ exists as a mix of monomers and dimers [30]. The crystal structure of EcAAC(6′)-Ib in complex with AcCoA and kanamycin C revealed that the active site is located within the monomer (Figure 3C) [25]. The active-site residues equivalent to Q106 and L107 of EcAAC(6′)-Ib are substituted in SeAAC(6′)-Ib_11_ with leucine and serine, respectively, which results in a wider substrate binding site that can accommodate larger aminoglycoside antibiotics. The EcAAC(6′)-Ib homolog from *P. aeruginosa* follows an ordered sequential kinetic mechanism where AcCoA binds to the active site first, followed by the aminoglycoside substrate. The EcAAC(6′)-Ib structure contains conserved features such as the “P-loop” L124-G125-K126-G127-L128-G129 and the V-shaped AcCoA binding cleft [25]. Differences with other members of the GNAT superfamily include the additional β7 strand that is placed between the β5 and β6 strands and a lack of the β-bulge. Detailed analysis of the EcAAC(6′)-Ib structure suggested that the essential residue D115 [25,31] could act as a general base to accept a proton in the AAC-catalyzed reaction, while the main-chain amide of Q116 forms a hydrogen bond with the carbonyl group of AcCoA, which is likely to stabilize the tetrahedral intermediate. The semi-conserved Y164 was proposed to act as a general acid either directly or via a conserved water molecule [25]. Recently, Chiem and colleagues [32] reported identification of an EcAAC(6′)-Ib inhibitor (1-[3-(2-aminoethyl)benzyl]-3-piperidin-1-ylmethyl)pyrrolidin-3-ol) by an in silico docking approach. The inhibitor targets the kanamycin A binding site. In addition, this compound could prevent the growth of an amikacin-resistant *Acinetobacter baumannii* clinical strain, suggesting that it also inhibits *A. baumannii* AAC(6′)-Ib [32].

The aminoglycoside 6′-*N*-acetyltransferase-Ie of *Staphylococcus warneri* (SwAAC(6′)-Ie-APH(2′′)) is a bifunctional enzyme which has an N-terminal *N*-acetyltransferase domain (AAC) and a C-terminal aminoglycoside phosphotransferase (APH) domain [24]. It confers resistance to nearly all commonly prescribed aminoglycoside antibiotics [26]. The 179-residue recombinant acetyltransferase domain crystallizes as a monomer. Structural analysis of this domain in complex with a sulfinic acid form of CoA showed that the CoA molecule is bound at the conserved V-shaped cleft formed by two β-strands splayed apart [24] (Figure 3D). SwAAC(6′)-Ie-APH(2′′) has no β-bulge at the active site and possesses an atypical glycine rich “P-loop” W108-S109-K110-G111-I112-G113 that interacts with the diphosphate arm of CoA. The aminoglycoside binding site is located in a highly negatively charged pocket formed by residues from the β4, β5 and β6 strands [24].

The chromosomally encoded 20.7 kDa 6′-*N*-acetyltransferase-Ii from *E. faecium* (EfAAC(6′)-Ii) has a broad substrate specificity for aminoglycosides [33]. In addition, it can acetylate histones and other small basic proteins [13]. The EfAAC(6′)-Ii is a dimer both in solution and in the crystal [34]. Structural analysis revealed that EfAAC(6′)-Ii has a β-bulge in strand β4 (residue H74 and P75), an atypical “P-loop” R83-K84-N85-Q86-I87-G88 and a V-shaped binding site between the β4 and β5 strands which are splayed apart to accommodate AcCoA (Figure 3E). Like EcAAC(6′)-Ib and SeAAC(6′)-Ib_11_, EfAAC(6′)-Ii has an additional strand (β7) located between the β5 and β6 strands. The hydroxyl group of Y147 forms a hydrogen bond with the sulfur atom of AcCoA. Kinetic analysis of EfAAC(6′)-Ii variants showed that Y147 does not act as a general acid in catalysis; instead, it is thought to be important for maintaining an optimal orientation of the acetyl group for efficient transfer [35]. EfAAC(6′)-Ii follows an ordered Bi-Bi mechanism (two on, two off) in which AcCoA binds to the enzyme first, followed by the aminoglycoside substrate [35,36]. Aminoglycoside-CoA bisubstrate analogs inhibit EfAAC(6′)-Ii at nanomolar concentrations, with some of them showing antimicrobial activity both in vitro and in vivo [37,38]. Analysis of their structure-activity relationships revealed that the inhibitory activity does not require the presence of the adenosine diphosphate (ADP) moiety, or the second or third aminoglycoside ring. At least one phosphate group is needed, although the pyrophosphate moiety can be replaced with β-dicarbonyl groups [37,38].

The chromosomally encoded aminoglycoside 6′-*N*-acetyltransferase-Iy from *S. enterica* (SeAAC(6′)-Iy) can catalyze 6′-*N*-acetylation of a wide range of 4,6- and 4,5-disubstituted aminoglycosides [22]. The 145-amino-acid SeAAC(6′)-Iy is a dimer both in solution and in the crystal [39]. It has a β-bulge in the β4 strand (residues E79 and G80), a typical “P-loop” (R88-Q89-R90-G91-V92-A93) and a V-shaped AcCoA binding site. The dimer interface has an exchanged β6 strand (Figure 3F)—a feature also found in *S. cerevisiae* histone *N*-acetyltransferase. Structural analysis of the SeAAC(6′)-Iy/CoA/ribostamycin ternary complex revealed that the 6′ amino group of ribostamycin is positioned close to the sulfur atom (~3.5 Å) of CoA, which would favor the direct acetyl transfer reaction. D115 on the β5 strand forms a water-mediated hydrogen bond with the 6′-amino group of the antibiotic molecule and was therefore proposed to serve as a general base in catalysis. No specific residue that could act as a general acid in this reaction was identified; a water-mediated protonation mechanism was proposed instead [39]. It follows a random sequential, rather than ordered, kinetic mechanism [22]. Structural and kinetic analysis of SeAAC(6′)-Iy showed that aminoglycoside-CoA bisubstrate analogs inhibit the enzyme at micromolar concentrations by binding to its active site in a mode similar to that observed for CoA and ribostamycin in the crystal structure of the SeAAC(6′)-Iy-CoA-ribostamycin complex [22].

Eis is a 45 kDa protein that enhances survival of *Mycobacterium* sp. within host cells by suppressing the host defense mechanisms [19,20]. Unlike other AACs, Eis and its homologs can acetylate multiple amines of aminoglycosides [19,40,41]. For example, Eis can transfer an acetyl group both on the 6′- and 3-amine group of kanamycin, and on the 3- and 4-amino-2-hydroxybutyryl group of amikacin [19]. In addition, Eis can use peptides as substrates. Eis exists as a hexamer both in solution and in the crystal [42], where two three-fold symmetrical trimers assemble into an asymmetric “sandwich”. Structural analysis of Eis in complex with CoA and acetylated hygromycin revealed that each Eis monomer harbors an N-terminal GNAT domain (residue 1–150), a central GNAT domain (residue 151–291) and a C-terminal domain with an atypical fold of the animal sterol carrier protein lacking a hydrophobic cavity (residues 292–402) (Figure 3G). Only the N-terminal GNAT domain is catalytically active; the central, inactive, GNAT domain lacks the conserved Gln/Arg residue in the “P-loop” that in other GNAT enzymes participates in recognition of the CoA pyrophosphate moiety. Aminoglycosides bind in the negatively charged pocket formed between the N-terminal and central GNAT domains [42]. The main-chain amino group of H119 plays an important role in proper positioning of the acceptor substrate, while the carbonyl oxygen of AcCoA is hydrogen-bonded with both F84 and V85, which might contribute to the stabilization of the tetrahedral reaction intermediate [42]. F402 could serve as a remote base that extracts a proton from the amino group of the acceptor substrate via a water molecule. The conserved Y126 residue is ~3.4 Å away from the sulfhydryl group of CoA and could serve as a general acid that donates a proton to the leaving thiolate anion. A mutagenesis study confirmed the presence of one catalytically active GNAT domain in Eis and the role of the Y126 residue as a general acid [42]. Comparison of the crystal structures of Eis proteins from *M. tuberculosis* (MtEis) and *Mycobacterium smegmatis* (MsEis) [41] revealed that the substrate binding site of MtEis is located in a deep narrow channel, where MsEis has a deep round pocket [41]. This is consistent with the endo-type peptide acylation activity of MtEis (on the Lys side chain) and the exo-type activity of MsEis (on the N-terminal amino group of peptides).

### 2.2. Histone N-Acetyltransferase Family (HATs, EC 2.3.1.48)

Histone N-acetyltransferases (HATs) acetylate the side chains of the conserved lysine residues on the N-terminal tail of histone proteins to promote transcriptional activation. HATs play a crucial role in chromatin remodeling and gene expression in eukaryotes [43]. HATs are classified into two types based on their subcellular localization and substrate specificities: (i) type A HATs which are present only in the nucleosome and can modify histones that are incorporated into chromatin; and (ii) type B HATs which are present in both the cytoplasm and nucleosome, and can acetylate only soluble free histones [43,44].

**Figure 4:**
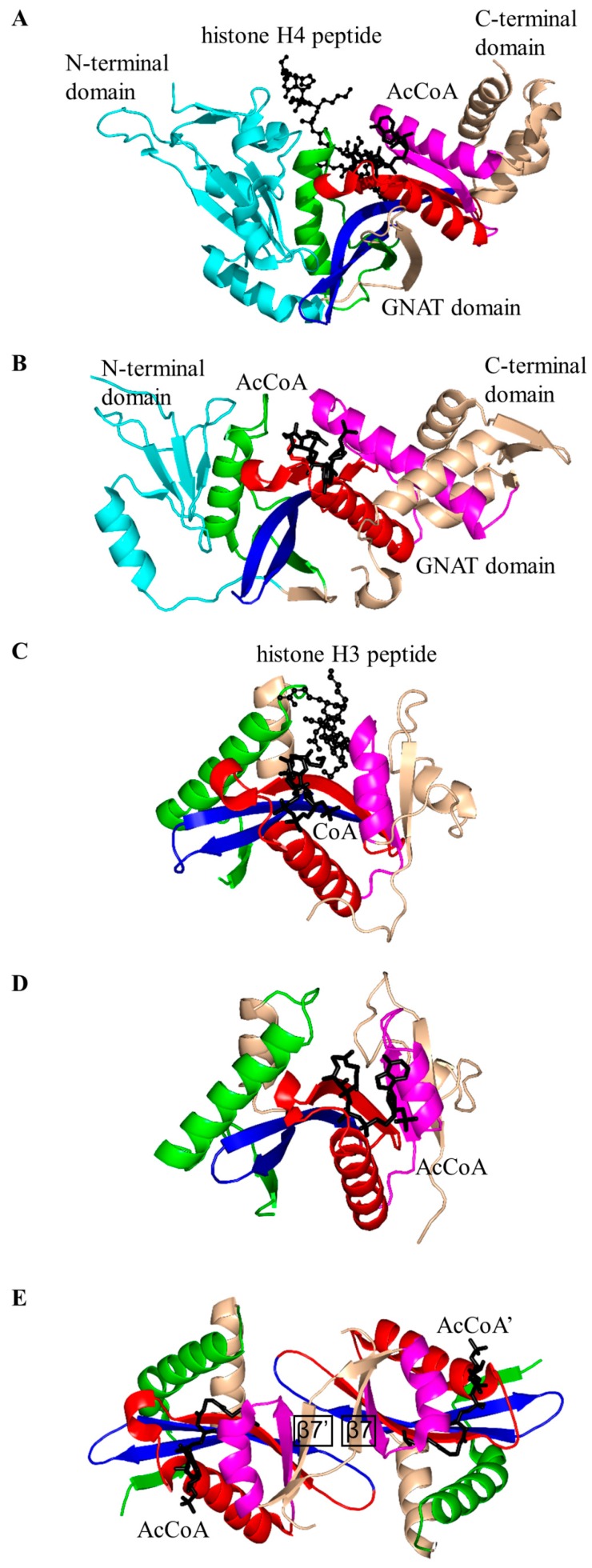
Cartoon representation of the structures of histone *N*-acetyltransferases. (**A**) Human HAT1 in complex with AcCoA and a histone H4 peptide (PDB ID: 2P0W [45]); (**B**) yeast Esa1 in complex with AcCoA (PDB ID: 1MJB [57]); (**C**) yeast GCN5 acetyltransferase in complex with CoA and a histone H3 peptide (PDB ID:1QSN [70]); (**D**) the histone acetyltransferase domain of hPCAF in complex with CoA (PDB ID: 1CM0 [73]); and (**E**) yeast Hpa2 histone acetyltransferase in complex with AcCoA (one dimer of the tetramer) (PDB ID: 1QSM [76]). In HAT1 and Esa1, the N-terminal domain is colored cyan, C-terminal domain is colored wheat, and conserved and non-conserved motifs of the central GNAT domain are colored as in Figure 1.

Histone *N*-acetyltransferase 1 (HAT1), found in *S. cerevisiae* and human, is type B HAT that acetylates the K5 and/or K12 residue of soluble free histone H4 [6,45]. Acetylation of histones by HAT1 might play an important role in chromatin assembly and DNA repair [44,46]. Several studies reported that there is a link between HAT1 expression levels and cancer [44]. Structural analysis of the ternary complex of human HAT1 (hHAT1) with AcCoA and histone H4 peptide revealed that hHAT1 has three domains: an N-terminal domain (residues 23–136), a central GNAT domain (residues 137–270), and a C-terminal domain (residues 271–341) (Figure 4A). The AcCoA binds in the cleft between the central and C-terminal domains [45]. The central domain has a glycine-rich “P-loop” that interacts with the pyrophosphate arm of AcCoA. The acetyl moiety of AcCoA is anchored by the interactions with I186, P278 and Y282 such that the acetyl group is placed close to the side chain of K12 of the histone H4 peptide, with a distance of ~4.3 Å between its carbonyl carbon and the ε-amino group of K12 [45]. The histone peptide is accommodated between the central and N-terminal domains. The conserved residues E64 and T199 of the N-terminal domain play an important role in the recognition of the acceptor substrate of hHAT1 [45]. Mutagenesis data confirmed that the combined action of the active-site residues E187, E276 and D277 deprotonates the ɛ-amino group of reactive K12 of the histone 4 peptide via a water molecule to facilitate the direct nucleophilic attack on the thioester acetate of AcCoA [45]. The structural homolog of hHAT1 in *S. cerevisiae* (ScHAT1) has an almost identical overall structural topology, except ScHAT1 has a longer β12–β13 loop, and a shorter β12 strand and β11–β12 loop [6].

The *S. cerevisiae* Esa1 (ScEsa1), type A HAT, is the sole histone acetyltransferase expressed in budding yeast [47]. ScEsa1 can acetylate histones H4, H2A and H2AZ [48,49,50,51,52,53]. It plays an important role in the regulation of gene expression and DNA repair [54,55,56]. The 445-amino-acid ScEsa1 has a central GNAT domain (residue 272–329) flanked by an N-terminal and C-terminal subdomains (Figure 4B) [57,58]. Structural analysis of ScEsa1 in complex with AcCoA showed that motif D is missing, and that AcCoA binds between the core GNAT domain and the C-terminal subdomain. Mutagenesis studies and structural analysis of a ScEsa1 complex with a bisubstrate inhibitor H4K16CoA, in which CoA is covalently linked to the side-chain amino group of the acetyl lysine residue in the histone H4 peptide substrate, revealed that autoacetylation of a conserved lysine (K262) in ScEsa1 contributes to substrate binding and acetylation [59]. Autoacetylation of ScEsa1 is also essential for the viability of yeast cells. Detailed analysis of the active site of ScEsa1 showed that E338 (equivalent to E579 in hPCAF and E255 in ScHat1) could act as a remote general base to extract a proton from the acceptor substrate [60]. Mutagenesis study confirmed its role in the acetyl transfer reaction [58]. Although the observation of an acetyl-cysteine covalent adduct in the crystals of ScEsa1 previously led to a hypothesis that this enzyme may employ a ping-pong reaction mechanism [57], subsequent kinetics analysis disproved that hypothesis and showed that ScEsa1 follows a direct nucleophilic attack mechanism [60].

Histone acetyltransferase of *S. cerevisiae* GCN5 (ScGCN5) is type A HAT that preferentially acetylates histones H3 (on K14) and H4 (on K8 and K16), but can also acetylate non-histone chromatin proteins including Spt2 [61]. ScGCN5 plays an important role in the control of expression of cell cycle-related and apoptosis-related genes [62]. It was first identified as an essential gene for biosynthesis of amino acids in yeast [63]. In addition, the *GCN5* null mouse showed increased apoptosis and mesodermal defects during embryo development [64]. Structural analysis revealed that ScGCN5 has a β-bulge in strand β4 (A124 and F125) and a conserved “P-loop” in motif A (Q133-V134-R135-G136-Y137-G138) that contributes to the AcCoA binding (Figure 4C) [65]. Structural analysis suggested, and mutagenesis and kinetics studies confirmed, that a conserved glutamate residue (E173) at the bottom of the putative substrate binding cleft serves as a general base to extract a proton from the ε-NH_2_ group of the lysine side chain on the substrate [65]. Structures of GCN5 homologs from other sources were subsequently solved, including those from human [66] and *Tetrahymena thermophila* [67,68,69,70]. Analysis of *T. thermophila* GCN5 (TtGCN5) in free form, in complex with AcCoA and in complex with CoA and a histone H3 peptide revealed a conserved sequence motif (G-K-X-P) that determines the histone-binding specificity [67,68,69,70]. The backbone NH group of L126 forms a hydrogen bond with the carbonyl oxygen of the acetyl group of AcCoA. L126 is therefore thought to play a crucial role in the reaction mechanism by polarizing the carbonyl group of the thioester and stabilizing the reaction intermediate [68]. TtGCN5’s conserved residue E122 (equivalent to residue E173 of ScGCN5) might act as a general base via a well-ordered water molecule to facilitate the direct acetyl transfer [67,68]. In the human homolog, the E575 residue, equivalent to residue E173 of ScGCN5, could serve as a general base to deprotonate the substrate [66]. Interestingly, the crystal structure of TtGCN5 bound to a peptide-CoA bisubstrate inhibitor suggested that after lysine acetylation, the H3 histone product is displaced from the substrate binding site through a rearrangement within the C-terminal domain of the enzyme [69].

The human p300/CBP-associating factor (hPCAF) facilitates activation of transcription by acetylating histone proteins in the nucleosome and other transcriptional activators, including the tumor protein p53 [71]. hPCAF (832 aa) is type B HAT that mainly acetylates K14 of histone H3. It also specifically acetylates p53 at K320 to promote its response to cellular DNA damage [72]. Structural and biochemical analysis of hPCAF in complex with CoA revealed that hPCAF exists as a dimer both in solution and in the crystal [72,73]. It has three domains: an N-terminal domain with limited homology to mammalian GNAT enzymes (1–492) [74], a central, catalytic HAT domain (residues 493–653) that shares a high degree of sequence homology with other GNAT histone acetyltransferases, and a C-terminal bromodomain (residues 725–819) (Figure 4D). The catalytic domain has a “P-loop” Q581-V582-K583-G584-Y585-G586 and a β-bulge in the β4 strand (residues V572 and F573). The CoA is bound in the V-shaped cleft between the β4 and β5 strands. Detailed analysis of the substrate binding site revealed that E570 (equivalent to E173 in ScGCN5) is optimally positioned to serve as a general base to extract a proton from the lysine residue of the substrate via a water molecule. The main-chain amide nitrogen of C574 forms a hydrogen bond with the carbonyl oxygen of AcCoA, that could stabilize the tetrahedral reaction intermediate [73].

The *S. cerevisiae* Hpa2 (ScHpa2) is type A HAT that acetylates the ε-amino group of lysine residues of histones H3 (K4 and K14) and H4 (K5, K12 and K8) [75]. In addition, ScHpa2 can acetylate non-histone chromosomal proteins (e.g., non-histone proteins Nhp6A and Nhp6B, high mobility group proteins Hmo1 and Hmo2) and polyamines, including putrescine, spermine and spermidine [75]. ScHpa2 (156 aa) exists as a dimer in solution. However, the ScHpa2/AcCoA binary complex is tetrameric both in solution and in the crystal [76]. This tetramer is a dimer of dimers, in which the adenine group of the AcCoA bound to one dimer interacts with the adenine group of the second AcCoA molecule bound to another dimer. The two ScHpa2 monomers assemble into a strand-swapped dimer shown in Figure 4E, where the β7 strand of each monomer is inserted between the β5 and β6 strands of the opposite monomer. ScHpa2 has a β-bulge in the β4 strand (residues N91 and D92) and an atypical “P-loop” V101-K102-G103-A104-G105-G106. Y139, the hydroxyl group of which forms a hydrogen bond with the sulfur atom of AcCoA, was proposed to serve as a general acid in the reaction. Mutagenesis of the equivalent Tyr residue (Y168) in spermidine/spermine N1-acetyltransferase (SSAT) confirmed its role in catalysis [77]. The positive charge around the entrance to the active site of ScHpa2 favors a deprotonated state of the ε-amino group of the substrate, thus negating a requirement for a specific general base residue [76].

### 2.3. Non-Histone Protein N-Acetyltransferase Family

The GNAT superfamily includes many enzymes that acetylate non-histone proteins. Protein acetyltransferase from *Sulfolobus solfataricus* (SsPAT) [78], human α-tubulin acetyltransferase 1 (αTAT1) [79], human Naa50p [80] and *M. tuberculosis* AcCoA synthetase *N*-acetyltransferase (Rv0998) [81] are representative members of this family, the structures of which have been characterized. Non-histone protein acetylation plays an important role in regulation of mRNA and protein stability, subcellular localization and breakdown of proteins and modulation of protein-protein and DNA-non-histone protein interactions [82,83].

**Figure 5:**
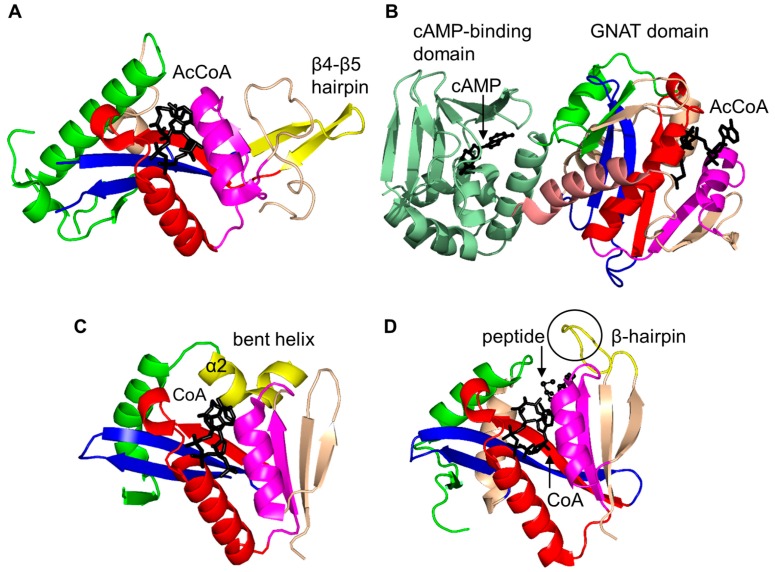
Cartoon representation of the structures of non-histone protein *N*-acetyltransferases. (**A**) Human α-tubulin acetyltransferase in complex with AcCoA (PDB ID: 4GS4 [79]); (**B**) *M. tuberculosis* MtPat in complex with cAMP and AcCoA (PDB ID: 4AB [81]); (**C**) *N*-acetyltransferase from *Sulfolobus solfataricus* in complex with CoA (PDB ID: 3F8K [78]); and (**D**) Human *N*-acetyltransferase Naa50p in complex with CoA and peptide MLGPEGGRWGRPVGRRRRP (PDB ID: 3TFY [80]). The conserved and non-conserved motifs of the GNAT domains, cofactors and substrates are colored as in Figure 3.

Acetylation of α-tubulin (on the ε-amino group of K40) by α-tubulin *N*-acetyltransferase 1 (αTAT1, EC 2.3.1.108) is involved in various microtubule-based processes in humans [79], mice [84] and other organisms, including *Caenorhabditis elegans* [85]. Structural analysis of 240-amino-acid human αTAT1 in complex with AcCoA showed that it has a “P-loop” Q131-R132-H133-G134-H135-G136 and a β-bulge involving L122 and D123 in the β4 strand [79]. The central β sheet is splayed apart to form a V-shaped AcCoA binding site (Figure 5A). Interestingly, αTAT1 has an additional 12-residue β-hairpin structure between strands β4 and β5, which forms part of the α-tubulin binding site (Figure 5A). Detailed analysis of the active site revealed that the conserved D157 and/or C120 residue could serve as a general base in the reaction. Mutagenesis studies confirmed the essential role of these two residues in catalysis [79]. An acetyl transfer mechanism involving two general base residues was also suggested for aralkylamine *N*-acetyltransferase (H120 and H122) [86] and Naa50p (H112 and Y173) [80]. The bisubstrate kinetics study showed that the reaction catalyzed by αTAT1 follows a ternary complex mechanism that does not involve a covalent protein/substrate intermediate [79]. The crystal structures of αTAT1 homologs from different species, including αTAT1 from mice [84] and Mec17 from human [87], were recently reported.

In *M. tuberculosis*, fusion of a cyclic nucleotide binding domain with a GCN5-like catalytic PAT domain within Rv0998 (also known as MtPatA) enables direct cAMP control of protein acetylation, whereupon cAMP binding allosterically regulates the PAT activity [88]. Although the physiological substrate of MtPatA is yet to be identified, it was shown that its ortholog from *M. smegmatis* acetylates both *M. smegmatis* and *M. tuberculosis* acetyl-CoA synthetase in vitro [89]. MtPatA comprises three domains: an N-terminal cNMP-binding domain (residues 12–142), a central catalytic GNAT domain (residues 146–314) and a C-terminal extension (residues 315–333). MtPatA was crystallized as a monomer (Figure 5B) [81]. Structural analysis of MtPatA in complex with AcCoA and cAMP revealed that cAMP binds at the N-terminal regulatory domain, AcCoA binds in the V-shaped cleft at the central catalytic domain, and the C-terminal extension serves as a regulatory element [81]. Kinetic and mutagenesis studies confirmed that E235 could serve as a general base, while R184 decreases the p*K_a_* of the ε-amino group of the Lys side chain of the substrate and thereby plays an important role in catalysis [81]. In contrast to other members of the non-histone protein *N*-acetyltransferase family, MtPatA can also efficiently catalyze transfer of alternative acyl groups including propionyl-CoA and butyryl-CoA.

Protein lysine acetyltransferase of *S. solfataricus* (SsPAT, EC 2.3.2.-) catalyzes the acetylation of the K16 residue of the archaeal DNA-binding protein ALBA (acetylation lowers binding affinity) to lower its binding capacity as a means of chromatin regulation [78]. Structural analysis of SsPAT in complex with CoA showed that the cofactor molecule binds in the V-shaped cleft between strands β4 and β5 such that its diphosphate arm interacts with the enzyme’s “P-loop” [78]. As with EcAAC(6′)-Ib, SeAAC(6′)-Ib_11_ and EfAAC(6′)-Ii, the β7 strand of SsPAT is placed between the β5 and β6 strands [21,25,30] (Figure 5C). Interestingly, SsPAT has a unique structural feature (a “bent helix” comprised of residues 32–41) that might be involved in auto-regulation of the enzyme’s acetyltransferase activity. Detailed analysis of the active site revealed that there is no single residue that could solely serve as a general acid or base in the reaction catalyzed by SsPAT [78], although mutagenesis studies suggested that a set of well-ordered residues (Y38, E42, E43, D53, H72 and E76) might serve as a “proton wire” to shuttle a proton from the active site [78].

Human *N*(α)-acetyltransferase Naa50p (hNaa50p, EC 2.3.2.-) transfers an acetyl group onto the α-amino group of the N-terminal methionine residue in proteins to regulate genome integrity [90]. It has a β-bulge in strand β4 (residues M75 and T76) and a typical “P-loop” R84-R85-L86-G87-I88-G89 that interacts with the pyrophosphate arm of CoA [80]. Structural analysis of the complex of hNaa50p with CoA and a peptide revealed that hNaa50p has a unique 14-residue β6–β7 hairpin that forms part of the substrate-binding site and determines the specificity for the N-terminal α-amino groups (Figure 5D). Mutagenesis, kinetic and structural studies suggested that Y73 and H112 can serve as the general base residues, deprotonating the amino group of the substrate via a water molecule, and that at least one of these residues can also act as a general acid in the reaction catalyzed by hNaa50p [80].

### 2.4. Arylalkylamine N-Acetyltransferase Family (AANAT, EC 2.3.1.87)

Arylalkylamine *N*-acetyltransferase (AANAT), also known as aralkylamine *N*-acetyltransferase or serotonin *N*-acetyltransferase, catalyzes transfer of an acetyl group from AcCoA to the primary amine of a wide range of arylalkylamine substrates, including dopamine, serotonin, phenylethylamine and tryptamine [91]. It catalyzes the penultimate step in the biosynthesis of melatonin from serotonin, and thereby controls coherent coordination of the sleep-wake cycle in vertebrates and seasonal reproduction in animals [91].

**Figure 6:**
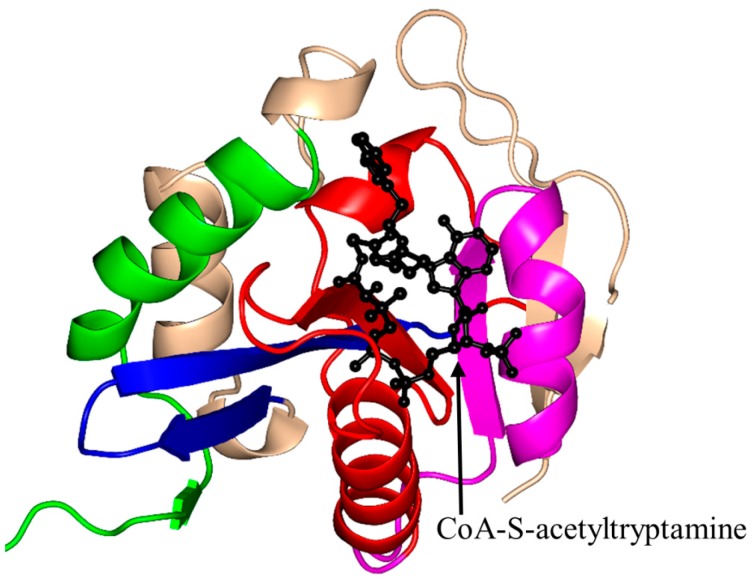
Cartoon representation of the structure of arylalkylamine *N*-acetyltransferase of *Ovis aries* in complex with a bisubstrate analog, CoA-*S*-acetyltryptamine (PDB ID: 1CJW [77]). The conserved and non-conserved motifs of the GNAT domain are colored as in Figure 3.

*Ovis aries* (sheep) AANAT (OaAANAT, 174 aa) exists as a monomer both in solution and in the crystal [77,86]. Structural analysis of OaAANAT in complex with a bisubstrate analog, CoA-*S*-acetyltryptamine, revealed that it has a conserved “P-loop” R131-Q132-Q133-G134-K135-G136 and a β-bulge at A123 and H122 in the β5 strand. The V-shaped cavity between the β4 and β5 strands accommodates AcCoA (Figure 6) [77,86]. The last β-strand (β7) is placed between the β5 and β6 strands. This structural feature is also found in some other GNAT enzymes [10]. The binding of AcCoA induces major structural rearrangements at the active site of OaAANAT that completes the serotonin binding pocket. This provided a structural basis for the ordered sequential mechanism where the binding of AcCoA at the active site of the enzyme is followed by the binding of the substrate tryptamine. The rate-limiting step in the acetylation reaction is the diffusional release of the product [92]. The Y168 is positioned appropriately to serve as a general acid, and its function was confirmed by mutagenesis studies [77]. Mutation of the Y168 to Phe resulted in a ~30-fold decrease of the *V*_max_ and a 27-fold increase in the *K*_M_ value of the enzyme [77].

Structures of the OaAANAT homologs from *Drosophila melanogaster* [93] and mosquito *Aedes aegypti* [94] have also been reported. Mosquito AANATs (aaNAT2, aaNAT5b and paaNAT7) have some unique features: the presence of helix/helices between the β3 and β4 strands, and a different active site residue (Val/Ala) corresponding to the conserved Y168 residue of OaAANAT [94]. Site-directed mutagenesis, kinetic studies and pH-rate profiling of *D. melanogaster* AANAT enzyme (DmAANAT) confirmed that it follows an ordered sequential Bi-Bi reaction mechanism where E47 serves as a remote general base that extracts a proton from the positively charged amino group of the acceptor substrate via a “proton wire” [93,95]. The substitution E47A resulted in a ~15-fold decrease of the *k*_cat,app_ value compared to wild-type [95].

### 2.5. Glucosamine-6-Phosphate N-Acetyltransferase 1 Family (GNA1, EC 2.3.1.4)

Glucosamine-6-phosphate *N*-acetyltransferase 1 (GNA1) catalyzes transfer of an acetyl group from AcCoA to the primary amine of d-glucosamine-6-phosphate (GlcN6P) to form *N*-acetylglucosamine-6-phosphate (GlcNAc-6P) [11]. The biosynthesis of GlcNAc-6P is a key step in the formation of the energy-rich metabolite UDP-*N*-acetyl-glucosamine (UDP-GlcNAc) [11,96]. UDP-GlcNAc serves as a precursor in the hexosamine biosynthesis pathway that is linked to the synthesis of major metabolites of glycolysis, lipid synthesis, tricarboxylic acid cycle and nitrogen cycle. In vertebrates, UDP-GlcNAc also acts as a precursor to generate UDP-*N*-acetylgalactosamine and cytosine monophosphate (CMP)-*N*-acetylneuraminic acid. In addition, UDP-GlcNAc serves as a substrate for chitin synthase and phosphatidylinositol-*N*-acetylglucosaminyltransferase (which catalyzes the first step in the biosynthesis pathway for glycophosphatidylinositol (GPI) anchors of various proteins) [96,97,98].

**Figure 7:**
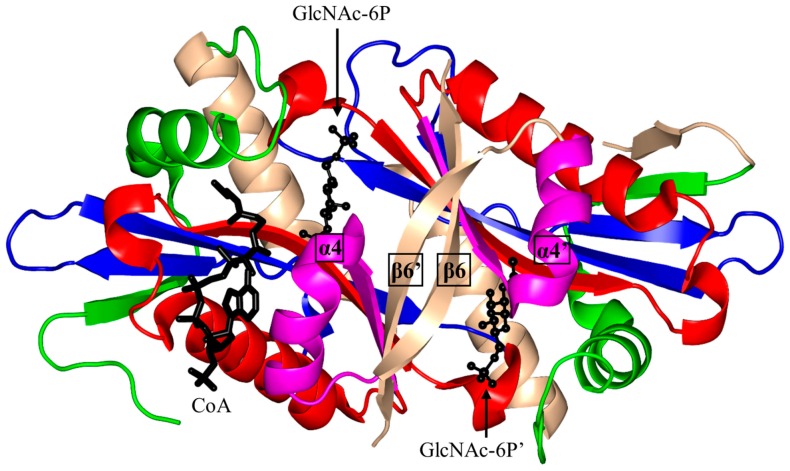
Cartoon representation of the structure of dimeric yeast glucosamine-6-phosphate *N*-acetyltransferase 1 in complex with CoA and *N*-acetyl-d-glucosamine-6-phosphate (GlcNAc-6P) (PDB ID: 1I1D [99]). The conserved and non-conserved motifs of the GNAT domain, cofactor and substrates are colored as in Figure 3.

Structural and biochemical analysis of *S. cerevisiae* GNA1 (ScGNA1, 161 aa) showed that ScGNA1 exists as a dimer both in solution and in the crystal (Figure 7) [99]. The two parallel β-strands (β4 and β5) of ScGNA1 are splayed apart to generate the characteristic V-shaped cleft that accommodates AcCoA. ScGNA1 has an atypical “P-loop” G108-Q109-G110-L11-G112-K113 that interacts with the diphosphate arm of AcCoA. It also has a β-bulge structure in strand β4 formed by residues D99 and I100 [99]. The β6 strand of each monomer is projected away from the core fold and forms part of the β-sheet of the opposite monomer, so that the two halves of the dimer are stabilized through “strand swapping”. C-terminal strand swapping was also observed in the structures of ScHpa2 and SeAAC(6′)-Iy [10,76]. Detailed structural analysis revealed that the binding site for the acceptor substrate is located at the dimer interface. The hydroxyl group of Y143 is within a hydrogen bonding distance from the sulfur atom of AcCoA, suggesting that Y143 may serve as a general acid and donate a proton to the leaving thiolate anion. Mutagenesis data supported the crucial role of Y143 for the activity of the ScGNA1 enzyme [11]. No candidates for a general base were identified in the ScGNA1 structure. However, the acceptor substrate in the reaction catalyzed by ScGNA1 (GlcN6P) has a much lower p*K_a_* (7.75) than that of other GNAT acceptor substrates and is likely to bind to the enzyme in a deprotonated form, eliminating the need for a general base [99]. The structures of the ScGNA1 homologs from *Homo sapiens* [100,101], *Aspergillus fumigatus* [100], *Arabidopsis thaliana* [102] and *C. elegans* [103] were also reported. The main-chain oxygen of E156 of human GNA1 (hGNA1) forms a hydrogen bond with the GlcN6P’s amino group and thus increases its nucleophilic nature. The essential role of E156 in the acceptor substrate binding and in catalysis was confirmed by mutagenesis [101].

### 2.6. Microcin C7 Self-Immunity Acetyltransferase Family (MccE)

Microcin C7 (McC) is a highly potent antibiotic, produced by certain *E. coli* strains, that can inhibit the growth of enteric bacteria by blocking aspartyl-tRNA synthetase and thus halting the cellular protein translation mechanism [104]. It is composed of heptapeptide MRTGNAD conjugated to modified AMP. The *E. coli* MccE protein acetylates McC converting it into a form that is non-toxic to the cell, thus conferring *E. coli* resistance to this compound [104,105].

**Figure 8:**
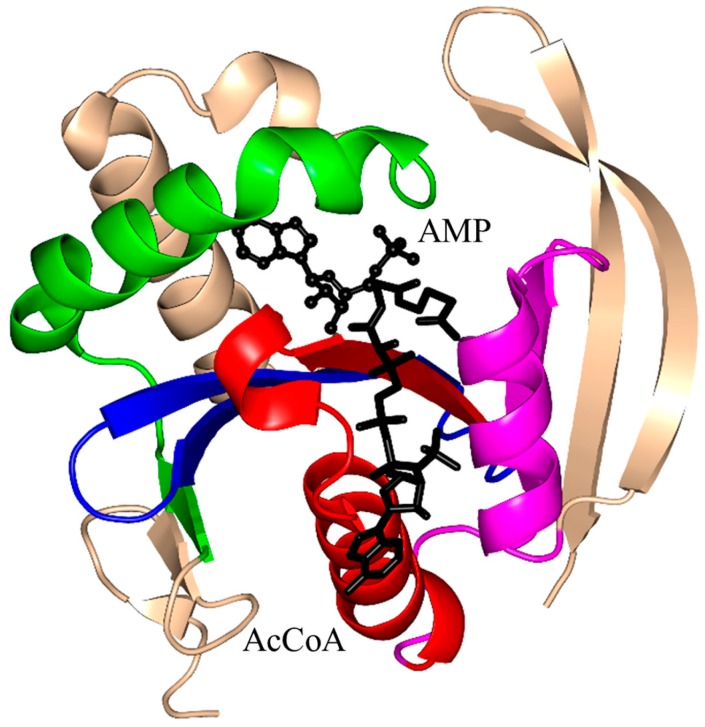
Cartoon representation of the structure of the acetyltransferase domain of *E. coli* MccE in complex with AcCoA and adenosine monophosphate (AMP) (PDB ID: 3R96 [104]). The conserved and non-conserved motifs of the GNAT domain, cofactor and substrate are colored as in Figure 3.

The acetyltransferase domain of MccE (188 aa) exists as a monomer both in solution and in the crystal [104]. The β4 and β5 strands are splayed apart to form the AcCoA binding site, and the last β7 is placed between the β5 and β6 strands (Figure 8). Unlike other GNAT proteins, MccE has an additional 70 amino acid residues at its N-terminus that form part of the substrate binding site (Figure 8). Structural analysis of MccE in complex with AcCoA and synthetic sulfamoyl adenylate substrates (aspartyl-sulfamoyl adenosine and glutamyl-sulfamoyl adenosine) revealed that T453 and F466 form π-stacking interactions with the adenine ring of the substrate analog, and thus play an important role in the substrate specificity [104]. The carbonyl oxygen of AcCoA interacts with the main-chain amide group of Y510 (~3.1 Å) and backbone carbonyl of I508. Residues S553 and E572 were proposed to serve as a general acid and a general base in the MccE-catalyzed reaction, respectively. An S553A/E572A variant of MccE showed significantly reduced enzymatic activity (~25-fold), confirming their importance for catalysis [105]. Although the side chain of C546 was found disulfide-bonded to the CoA thiol in the crystal, mutagenesis data on MccE homologs and the lack of conservation of this residue rule out the possibility that MccE catalyzes acetyl transfer by a ping-pong mechanism [104].

### 2.7. Pseudaminic Acid Biosynthesis Protein H Family (PseH, EC 2.3.1.202)

Pseudaminic acid biosynthesis protein H (PseH) catalyzes the third step of the biosynthesis pathway for pseudaminic acid in *Helicobacter pylori* and *Campylobacter jejuni* to form UDP-2,4-diacetamido-2,4,6-trideoxy-β-l-altropyranose (UDP-sugar) from UDP-4-amino-4,6-dideoxy-β-l-AltNAc using AcCoA as an acetyl donor [106]. PseH plays an important role in flagella assembly and function. Mutation of the *pseH* gene in *C. jejuni* resulted in non-motile phenotype [106].

**Figure 9:**
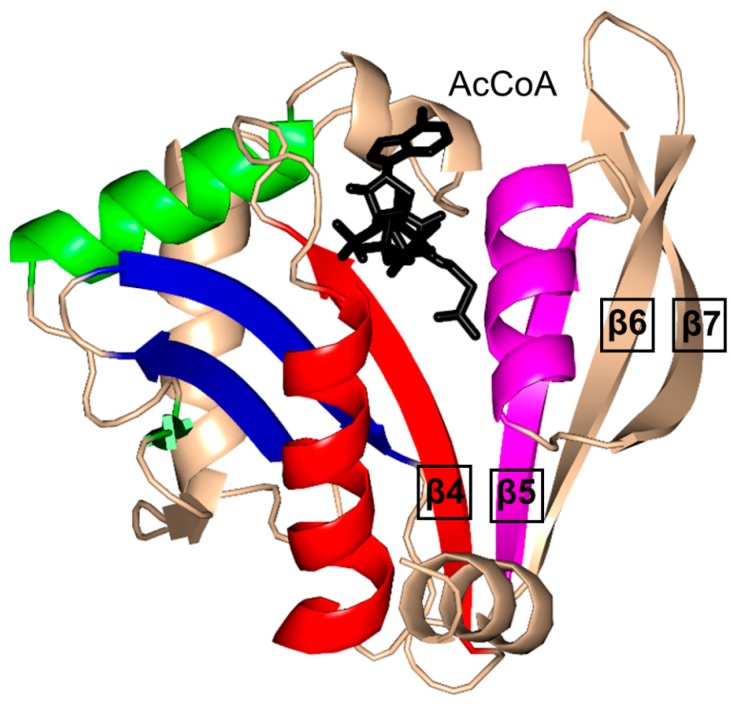
Cartoon representation of the structure of *Helicobacter pylori* pseudaminic acid biosynthesis protein H in complex with AcCoA (PDB ID: 4RI1 [107]). The conserved and non-conserved motifs of the GNAT domain are colored as in Figure 3.

*H. pylori* PseH (HpPseH, molecular weight (*M*_W_) 21.1 kDa) exists as a homodimer in solution, although there are three subunits of PseH in the crystal [107]. Two of these subunits form a non-crystallographic dimer similar to that found in the crystals of *S. typhimurium* RimL [108], while the third subunit forms a similar dimer with a symmetry-related subunit. Structural analysis of HpPseH in complex with AcCoA showed that the β4 and β5 strands are splayed apart, creating a channel through the molecule that accommodates AcCoA (Figure 9). The β7 strand is sandwiched between the β5 and β6 strands. It has an atypical “P-loop” that interacts with the pyrophosphate arm of AcCoA. The β-bulge structure is missing in HpPseH. Furthermore, it has an additional β-strand at the N-terminus and an additional α-helix at the C-terminal end. The putative catalytic site is located within each monomer. At the dimer interface, the C-terminal β-strands from each monomer form a continuous β-sheet similar to that present in EfAAC(6′)-Ii [34] and ScGNA1 [99]. The carbonyl oxygen of AcCoA is hydrogen-bonded to the main-chain amide of I93 and the OH group of Y138. Detailed analysis of the modeled ternary complex (HpPseH/AcCoA/UDP-sugar) showed that the side chain of the conserved S78 residue could act as a remote general base to extract a proton from the 4-amino group of UDP-sugar via a well-ordered water molecule. The structure also suggested that conserved Y138 could serve as a general acid in catalysis, consistent with the role of the corresponding residue in other GNAT proteins [107]. HpPseH and its homolog from *C. jejuni* (CjPseH) share many structural features [109], although in contrast to HpPseH, CjPseH exists as a monomer in solution and in the crystal [109].

### 2.8. Thymidine Diphosphate (TDP)-Fucosamine Acetyltransferase Family (WecD, EC 2.3.1.210)

*E. coli* TDP-fucosamine acetyltransferase (WecD, 235 aa) catalyzes the final step in the biosynthesis of an outer membrane glycolipid termed enterobacterial common antigen (ECA). It acetylates dTDP-4-amino-4,6-dideoxy-α-d-galactose to TDP-4-acetamido-4,6-dideoxy-d-galactose using AcCoA as an acetyl donor. ECA is widely distributed among Enterobacteriaceae and plays an important role in bacterial resistance to organic acids and intestinal bile salts used as a defense mechanism by the eukaryotic host [110,111]. It also plays a crucial role in flagella biosynthesis, and thus affects bacterial motility and virulence [112].

**Figure 10:**
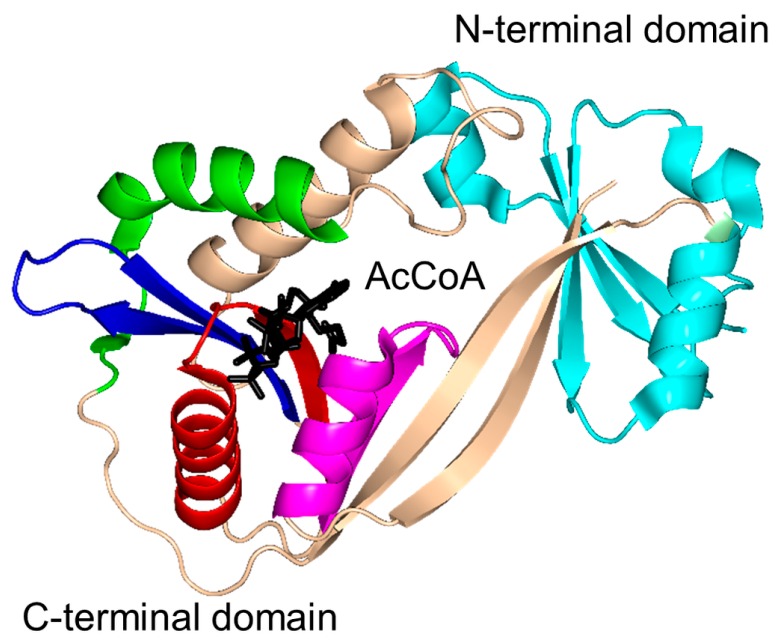
Cartoon representation of the structure of *E. coli* WecD in complex with AcCoA. The N-terminal partial GNAT domain is colored cyan (PDB ID: 2FT0 [113]). The conserved and non-conserved motifs of the GNAT domain are colored as in Figure 3.

WecD exists as a dimer both in solution and in the crystal [113]. Structural analysis of the WecD/AcCoA binary complex revealed that each monomer harbors a typical C-terminal GNAT domain (residues 70 to 218) and an N-terminal partial GNAT domain (residues 3 to 69, and 219 to 224) lacking the first two α-helices and two β-strands (Figure 10). The C-terminal domain contains a β-bulge involving G66 and L167 of the β8 strand, and an atypical “P-loop” A169-G170-R171-G172-A173-G174. AcCoA binds in the structurally conserved V-shaped cleft at the C-terminal GNAT domain. Detailed analysis of the modeled ternary complex WecD/AcCoA/TDP-fucosamine showed that the conserved Y208 residue would be within the hydrogen bonding distance (~3.0 Å) of the thioester moiety of AcCoA, and that the backbone carbonyl oxygen of A196 could form hydrogen bonds with the 4-amino group of the substrate. Like in most other GNAT enzymes, Y208 is believed to play an important role in the reaction by (i) ensuring the proper positioning of the acetyl group for transfer; and (ii) stabilizing and protonating the thiolate anion of the leaving CoA [113]. Mutation of the corresponding tyrosine residue in ScGNA1 and serotonin *N*-acetyltransferase confirmed its essential role in catalysis [11,77,86]. Although no residue in the immediate vicinity of the substrate was identified as a likely general base, the structural analysis suggested that E68, which was ~7.5 Å away from the 4-amino group in the modeled Michaelis complex, could extract a proton from the amino group of the substrate via water molecules.

### 2.9. Tabtoxin Resistance Protein Family (TTR, EC 2.3.1.-)

Tabtoxin resistance protein (TTR, *M*_W_ ~ 19 kDa) inactivates tabtoxin (the progenitor of the highly virulent non-specific bacterial toxin tabtoxinine β-lactam (TβL)) by acetylation, and thus gives self-immunity to tabtoxin-producing pathogens, such as *P. syringae* [114,115]. TβL is the predominant phytotoxin that causes the tobacco wildfire disease [114,115]. In addition, TβL can kill microbes and damage mammalian and plant cells by inhibiting glutamine synthetase that contributes to the neutralization of cellular ammonia [116]. Transgenic plants that produce TTR can prevent wildfire disease caused by *P. syringae* pv. *Tabaci* [117,118].

**Figure 11:**
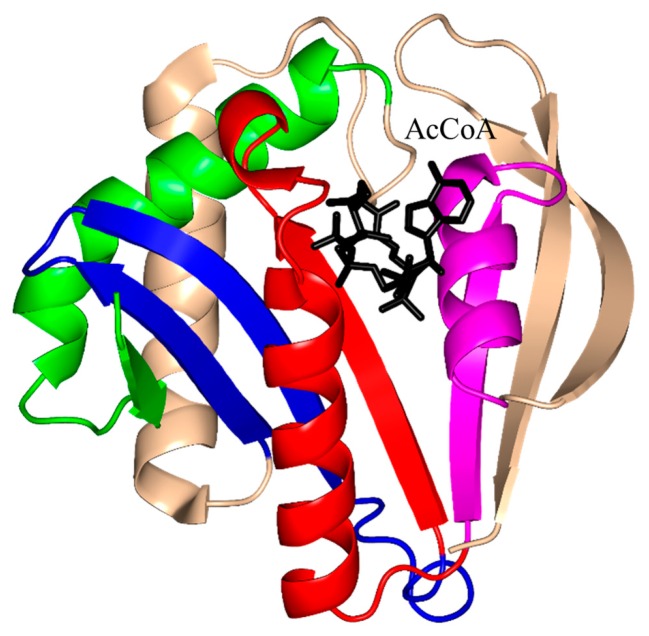
Cartoon representation of the structure of the tabtoxin resistance protein from *Pseudomonas syringae* in complex with AcCoA (PDB ID: 1GHE [114]). The conserved and non-conserved motifs of the GNAT domain are colored as in Figure 3.

Structural analysis of TTR in complex with co-purified AcCoA showed that this protein exists as a dimer in the crystal [114]. Like most other GNAT family members, TTR has a β-bulge, formed by residues Q94 and K95 on the β4 strand, and a characteristic V-shaped AcCoA binding site between strands β4 and β5 (Figure 11). It harbors the conserved six-residue sequence (Arg/Gln)-X-X-Gly-X-(Gly/Ala) in motif A that is responsible for the AcCoA recognition and binding. The binding mode of AcCoA is similar to most other GNAT enzymes [10,76]. The carbonyl group of AcCoA forms a hydrogen bond with the hydroxyl group of the conserved residue Y141. In the modeled ternary complex TTR/AcCoA/TβL, the imide nitrogen of TβL is positioned ~4.7 Å from the carbonyl carbon of AcCoA, while the imide carbon of TβL interacts with E92 and D130 via a well-ordered water molecule (4.6 Å). This water molecule might be crucial for the initial deprotonation and thus a water-mediated direct acetyl transfer mechanism was proposed for the TTR-mediated reaction. E92 and D130 are thought to act together as a general base in catalysis, whilst Y141 could serve as a general acid that donates a proton to the sulfur atom of CoA [114].

### 2.10. Mpr1 Family (EC 3.4.1.-)

Mpr1, a 229-residue antioxidant enzyme, encoded by the sigma 1278b gene for proline-analog resistance of *S. cerevisiae*, was initially reported as *N*-acetyltransferase that detoxifies the proline analog l-azetidine-2-carboxylate (AZC) by transforming it into *N*-acetyl-AZC [119]. In addition, Mpr1 is involved in l-proline metabolism by acetylating intermediate molecules, including l-Δ^1^-pyrroline-5-carboxylate (P5C) and l-glutamate-γ-semialdehyde (GSA), and thus plays an important role in defending yeast cells from oxidative stress [120,121,122]. While most GNAT enzymes acetylate primary amines, Mpr1 acetylates only cyclic secondary amines such as AZC and *cis*-4-hydroxy-l-proline (CHOP) [119].

**Figure 12:**
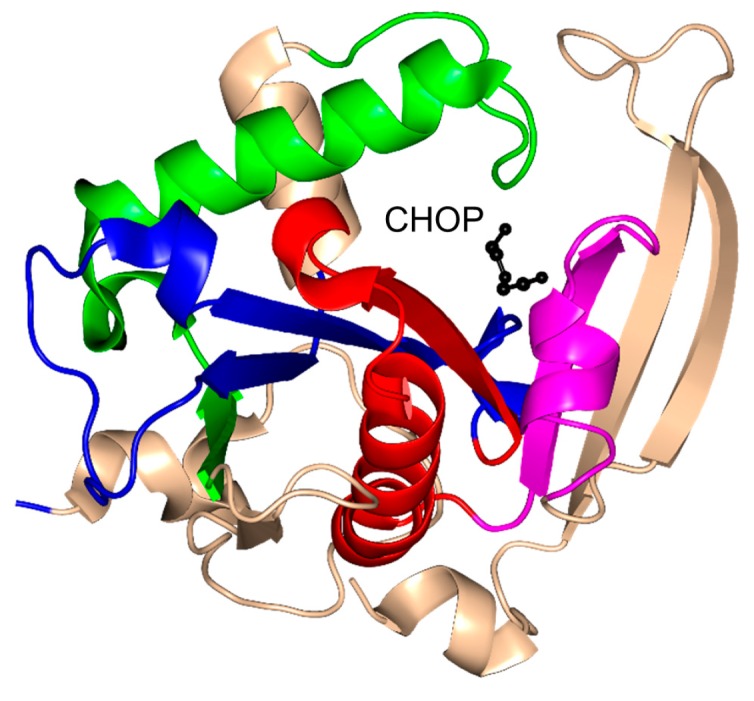
Cartoon representation of the structure of yeast Mpr1 in complex with *cis*-4-hydroxy-l-proline (CHOP) (PDB ID: 3W6X [119]). The conserved and non-conserved motifs of the GNAT domain are colored as in Figure 3.

Mpr1 exists as a dimer in solution [119]. Analysis of the crystal structure of Mpr1 in complex with its natural substrate CHOP revealed that the β-bulge is missing (Figure 12). In the modeled ternary complex Mpr1/CHOP/AcCoA, the sulfur atom of AcCoA interacts with the side chain of N178. Substitution of N178 with alanine decreased the *k*_cat_ ~40-fold, indicating that N178 is essential for catalysis [119]. The initial velocity pattern analysis revealed that the Mpr1-catalyzed reaction proceeds via a direct acetyl transfer mechanism that involves formation of a tetrahedral intermediate. The breakdown of this intermediate generates a thiolate anion which is stabilized by the interaction with the side-chain amide of N178, and then a water molecule bound to N178 donates a proton to release CoA. The use of a side-chain amide of an Asn residue in enzymatic catalysis is a unique feature of Mpr1.

### 2.11. Spermidine/Spermine N1-Acetyltransferase Family (SSAT, EC 2.3.1.57)

Spermidine/spermine *N*1-acetyltransferase (SSAT) is one of the enzymes in the polyamine degradation pathway [123]. It uses AcCoA as the donor substrate. Acetylation of spermidine/spermine promotes expulsion or breakdown of polyamines. Abnormally high cellular polyamine levels in human cells are associated with different diseases, including Alzheimer’s, cystic fibrosis and cancer [123,124,125].

**Figure 13:**
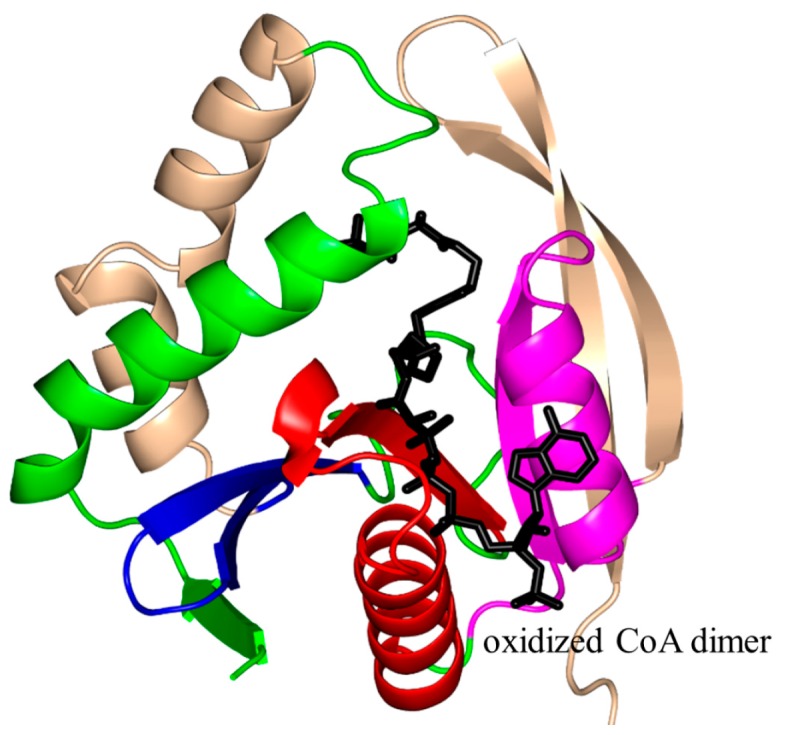
Cartoon representation of the structure of *Bacillus subtilis* PaiA in complex with an oxidized CoA dimer (PDB ID: 1TIQ [126]). The conserved and non-conserved motifs of the GNAT domain are colored as in Figure 3.

*Bacillus subtilis* PaiA (BsPaiA, 172 aa) is a member of the SSAT family that exists as a monomer in solution and a dimer in the crystal [126]. The structure of BsPaiA has the characteristic V-shaped groove for AcCoA binding (Figure 13). Interestingly, the structural elements that are likely to be involved in substrate binding (the loop connecting strands β3 and β4 and the C-terminal region comprising the β6 and β7 strands) show significant differences with other GNAT proteins, and are therefore thought to be important determinants of the substrate specificity of BsPaiA. One of the two subunits in the asymmetric unit of the crystal of the BsPaiA/CoA binary complex contained a single CoA molecule, and the other an oxidized CoA dimer. One of the monomers of this dimer occupies the natural AcCoA binding site, whereas the second CoA monomer is bound in the other end of the active-site tunnel, which is where the acceptor substrate is thought to bind. Structural analysis of BsPaiA showed that the side chain of Y142, that interacts with the sulfur atom of CoA, could serve as a general acid in catalysis. Kinetic analysis with the physiological substrate (spermine) showed that the enzyme follows Michaelis–Menten kinetics with an apparent *K_m_* of 76 µM, a *V*_max_ of 480 nmol/min/mg enzyme and a *k*_cat_ of 19.1 min^−1^ [126].

Analysis of the crystal structures of SSAT homologs from other sources including human, mouse, *Vibrio cholerae* and *Thermoplasma acidophilum*, revealed different oligomeric forms [127,128,129,130]. SSATs from human (hSSAT) and mouse (*Mus musculus*) (MmSSAT) form a dimer in the crystal by interchanging the β7 strands between the two monomers. Interestingly, the SSAT homolog from *V. cholerae* (SpeG) exists as a dodecamer (dimer of hexamers) both in solution and in the crystal [127]. However, each monomer of the hexamer forms a dimer with a neighboring monomer from the second hexamer. The way the two monomers associate into the dimer in SpeG is different to the SSAT homologs from human and mouse, but similar to many GNAT enzymes from other families, including EfAAC(6′)-Ii [13,34] and StRimL [10]. The SpeG dodecamer has six allosteric sites and six active sites [131]. Binding of a polyamine molecule (spermidine/spermine) to the allosteric site of SpeG induces structural changes that result in binding of AcCoA and an additional polyamine molecule to the active site [131].

The bell-shaped *k*_cat_ pH profile of hSSAT is consistent with the involvement of both a catalytic acid (with a p*K_a_* value of 8.9) and a catalytic base (with p*K_a_* of 7.3), whose ionization is crucial for catalysis [132]. Detailed structural analysis of the hSSAT/AcCoA binary complex revealed that the sulfur atom of AcCoA is within a hydrogen bonding distance from the conserved residue Y140, which serves as a general acid in catalysis [132]. Substitution of Y140 with phenylalanine lowered the enzyme’s activity to less than 5% of the wild-type and thus confirmed its role as a catalytic residue [133,134]. Analysis of hSSAT in complex with the bisubstrate *N*^1^-spermine-AcCoA showed that E92 could serve as a remote general base to perform water-mediated proton extraction from spermine [132]. Substitution of E92 with glutamine in the mouse homolog MmSSAT confirmed the essential role of E92 in the reaction [128]. An ordered sequential mechanism for acetyl transfer (polyamine binds first, followed by AcCoA) was observed in rat liver SSAT [128], whereas BsPaiA and hSSAT follow a random-order ternary complex mechanism [126].

### 2.12. C-Terminal N^ε^-Lysine Protein Acetyltransferase Family (EC 2.3.1.-)

*N^ε^*-lysine acetylation of proteins occurs in all domains of life [135]. Lysine acetylation contributes to the control of gene expression in eukaryotes, archaea and some bacteria. It plays an important role in bacterial metabolism and other aspects of cell physiology [135,136].

**Figure 14:**
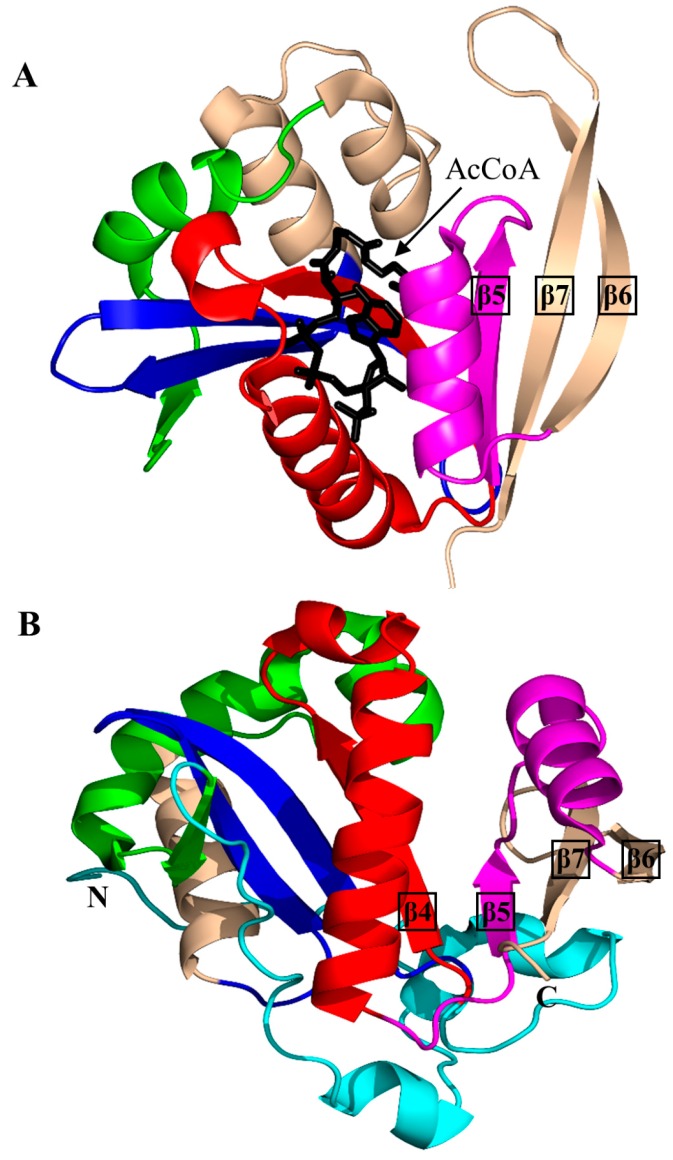
Cartoon representation of the structure of *N^ε^*-lysine protein acetyltransferase. (**A**) *Pseudomonas aeruginosa* PA4794 in complex with AcCoA (PDB ID: 3PGP [137]); and (**B**) Rv1347c of *M. tuberculosis* (PDB IS: 1NYK3 [138]). The conserved and non-conserved motifs of the GNAT domains are colored as in Figure 3.

PA4794 (160 aa) is C-terminal *N^ε^*-lysine protein acetyltransferase from *P. aeruginosa* that catalyzes both *N*- and *O*-acetylation of proteins or small molecules, including chloramphenicol [137]. PA4794, the structure of which is shown in Figure 14A, is monomeric both in solution and in the crystal [137]. It contains a conserved V-shaped cleft between the β4 and β5 strands, a typical “P-loop” at the N-terminal end of the α3 helix (R88-G89-L90-G91-V92-A93), and a β-bulge at A77 in the β4 strand. Structural analysis of the PA4794/AcCoA binary complex revealed that a tyrosine residue (Y128) is positioned near the thioester sulfur atom of AcCoA, and can therefore serve as a general acid by donating a proton to the leaving thiolate anion of CoA. The thioester oxygen of AcCoA forms a hydrogen bond with the backbone amide of M81 and thus contributes to the proper positioning and stabilization of AcCoA. Analysis of the ternary complex of PA4794 with CoA and acetylated peptide *N*-phenylacetyl-Gly-Lys revealed the role of the C-terminal free carboxyl group of the peptide in promoting the acetylation of lysine. There was no appropriately oriented conserved residue in the structure that could be a candidate for a general base. However, several well-ordered water molecules are present in the active site that form interactions with the main chain peptide groups of Y28 and F118, and the side chain of N121, and that can mediate extraction of a proton from the primary amine of an acceptor substrate. The Y128F and Y128A variants of PA4794 were catalytically inactive, which confirmed its essential role in the reaction. Furthermore, substitution of N121 with Ala resulted in a significant decrease in the enzyme activity [137], confirming its role in catalysis. In addition, structural analysis of PA4794 in complex with antibiotics showed that cephalosporins occupy the substrate-binding site and thus act as competitive inhibitors [137].

Rv1347c (210 aa) is lysine *N^ε^*-acyltransferase of *M. tuberculosis*. It is essential for the mycobacterial survival within infected cells and contributes to virulence [138]. Rv1347c is involved in the biosynthesis of the mycobactin siderophore in *M. tuberculosis*. It catalyzes acetylation of one or both of the *N^ε^*-hydroxylysine arms of mycobactin [138]. The biosynthesis of siderophores was also linked with pathogenesis in other bacteria [139,140,141,142]. Structural analysis revealed that Rv1347c crystallizes as a monomer [138]. It has a V-shaped AcCoA binding site and an atypical “P-loop”. Rv1347c has no β-bulge in the β4 strand. However, it harbors a catalytically important H130 residue at the corresponding β-bulge position [143]. It has an N-terminal extension that serves as a cap on top of the substrate binding cavity (Figure 14B). Another deviation from the conserved core GNAT fold in Rv1347c is that the β7 strand is positioned between the β5 and β6 strands. A kinetic study with a bisubstrate inhibitor and pH dependence assays showed that Rv1347c follows a random-order ternary complex mechanism in which H130 serves as a general base. D168 was shown to be also important for catalysis, although its specific role remains to be established [143].

### 2.13. Ribosomal Protein N^α^-Acetyltransferase Family (EC 2.3.1.128)

*N^α^*-acetylation of proteins is a common phenomenon in eukaryotes, where it plays an important role in the control of proteins function and stability [144]. In prokaryotes, where *N^α^*-acetylation is less common, transferases RimI, RimJ, and RimL acetylate the α-amino group of N-terminal amino acids in ribosomal proteins S18, S5 and L12, respectively [145,146].

**Figure 15:**
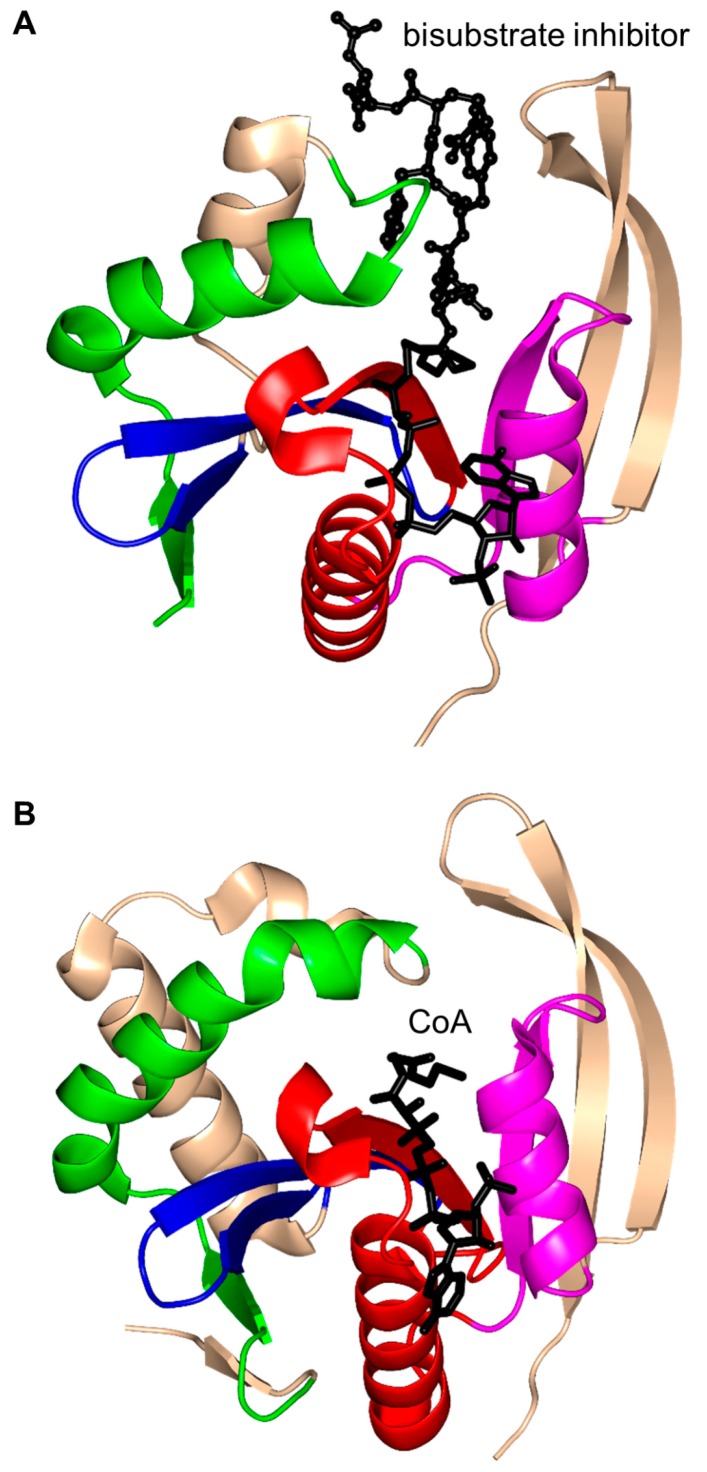
Cartoon representation of the structures of ribosomal protein *N^α^*- acetyltransferases. (**A**). *Salmonella typhimurium* RimI in complex with a bisubstrate inhibitor, C-term-Arg-Arg-Phe-Tyr-Arg-Ala-N-α-AcCoA (PDB ID: 2CNM [147]); and (**B**) RimL from *S. typhimurium* in complex with CoA (PDB ID: 1S7N [108]). The conserved and non-conserved motifs of the GNAT domains are colored as in Figure 3.

RimI (148 aa) of *S. typhimurium* (StRimI) exists as a monomer in solution; the monomers assemble into a trimer in the crystal, but this trimer does not appear to be physiologically relevant [147]. Structural analysis revealed that strands β4 and β5 of the core β-sheet form a V-shaped cleft, and the β7 strand is positioned between the β5 and β6 strands (Figure 15A). StRimI has a conserved “P-loop” Q76-R77-R78-G79-L80-G81 between the β4 strand and the α3 helix that interacts with the pyrophosphate moiety of AcCoA. The carbonyl moiety of AcCoA forms a hydrogen bond with the main-chain NH group of I69 that is thought to polarize the acetyl group and, in addition, play an important role in stabilizing the tetrahedral reaction intermediate. Detailed analysis of the structures of the StRimI/AcCoA and StRimI/bisubstrate analog complexes revealed that Y115 forms a hydrogen bond with the sulfur atom of AcCoA, and could therefore serve as a general acid that donates a proton to the leaving group. Furthermore, the structures suggested that E103 can serve as a general base that extracts a proton from the primary amine of the acceptor substrate via a well-ordered water molecule. Kinetic analysis showed that StRimI follows a direct ordered nucleophilic addition-elimination mechanism where AcCoA binds first, followed by the acceptor substrate [147].

RimL (179 aa) of *S. typhimurium* (StRimL) is responsible for converting the prokaryotic ribosomal protein L12 into its acetylated form (L7) by transferring an acetyl group from AcCoA onto the N-terminal amino group of L12 [108]. StRimL also possesses the MccE activity [148]. It is dimeric both in solution and in the crystal. Structural analysis revealed that the β4 and β5 strands of StRimL are splayed apart to create a V-shaped groove for AcCoA binding (Figure 15B). The “P-loop” between the β4 strand and the α3 helix interacts with the diphosphate group of CoA. The StRimL structure contains no β-bulge in the active site [108]. The β7 strand is placed between the β5 and β6 strands, and the β-strands at the dimer interface are arranged to form a continuous β-sheet. The dimer interface contains the protein substrate binding site. Kinetic analysis showed that StRimL follows a direct acetyl transfer mechanism [108]. The carbonyl oxygen of AcCoA is hydrogen-bonded with the main-chain amide of Y98 that could serve for the proper positioning of the acetyl group and for the polarization of the carbonyl carbon, and thereby stabilization of the tetrahedral reaction intermediate. S141 is within hydrogen-bonding distance from the sulfur atom of bound CoA, and can therefore serve as a general acid in catalysis. E160 is appropriately positioned to act as a general base to extract a proton from the α-amino group of L12 as the first reaction step [108]. The structures of RimL and its homolog from *B. subtilis*, YadF, are very similar [149]. However, in contrast to the dimeric RimL, YadF exists as a mixture of dimers and hexamers in solution and crystallizes as a trimer of dimers.

### 2.14. Succinyltransferase Family (EC 2.8.3.-)

**Figure 16:**
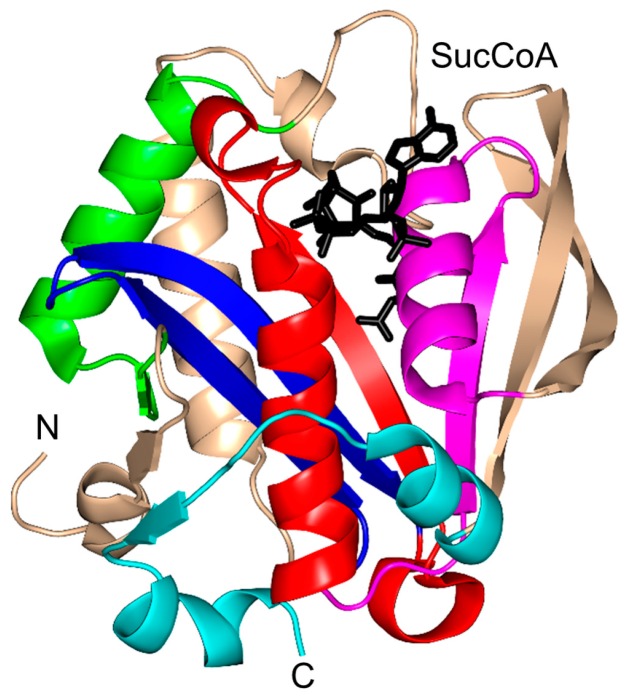
Cartoon representation of the structure of putative *M. tuberculosis* succinyltransferase (Rv0802c) in complex with succinyl CoA (PDB ID: 2VZZ [4]). The conserved and non-conserved motifs of the GNAT domain are colored as in Figure 3.

Rv0802c of *M. tuberculosis* (MtRv0802c, 218 aa) is a putative succinyltransferase that is thought to utilize succinyl CoA (SucCoA), rather than AcCoA, as an acyl donor [4]. Structural and biophysical analysis revealed that MtRv0802c exists as a tetramer (dimer of dimers) both in solution and in the crystal, as is the case for yeast Hpa2 [4,76]. Each monomer of MtRv0802c accommodates one molecule of SucCoA. It has a “P-loop” between the β4 strand and the α3 helix that forms hydrogen bonds with the pyrophosphate moiety of SucCoA. The SucCoA binds in the V-shaped cleft between the β4 and β5 strands (Figure 16). MtRv0802c has a C-terminal extension (two α-helices and a β-strand), not found in other GNAT proteins. However, the function of this extension is not yet known. Detailed analysis showed that the carbonyl oxygen of the succinyl group forms a hydrogen bond with the main-chain amide of S111 on the β4 strand. The hydrogen bond between the carbonyl group of SucCoA and the main-chain amide of a residue on the β4 strand is a conserved feature found in other GNAT proteins [10].

### 2.15. FemABX Aminoacyl Transferases Family (FemABX, EC 2.3.2.-)

Members of the FemABX (factors essential for methicillin resistance) family are involved in the synthesis of peptide bridges that crosslink peptides in peptidoglycan in some bacteria. They transfer an aminoacyl group from aminoacyl-tRNA to the ε-amino group of Lys or *meso*-diaminopimelic acid at position 3 of the peptidoglycan precursor, which can be either UDP-MurNAc-pentapeptide, or lipid II [150,151,152]. They play an important role in maintaining the peptidoglycan structure and thus contribute both to the bacterial viability and methicillin resistance [153,154]. FemX adds the first residue, FemA catalyzes the addition of the second and third residue (typically glycine) to the growing pentaglycine interpeptide, while FemB incorporates the fourth and fifth glycine [151]. Insertional inactivation of the *femAB* operon resulted in altered glycine composition in the bacterial cell wall [155]. The *femAB* knock-out strain showed absolute sensitivity to methicillin and, in addition, became hypersensitive to other types of antimicrobial agents [156].

**Figure 17:**
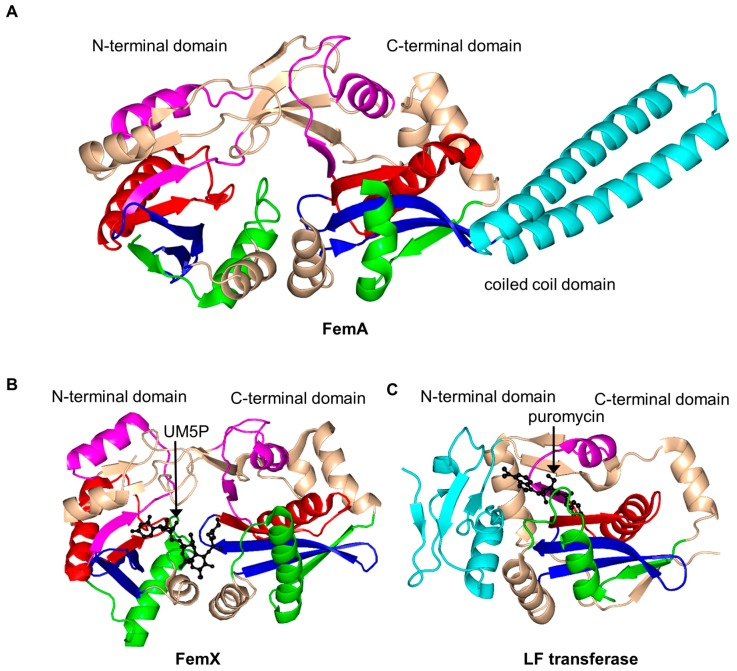
Cartoon representation of the structures of aminoacyl transferases from the FemABX family. (**A**) *Staphylococcus aureus* FemA apoenzyme (PDB ID: 1LRZ [150]); (**B**) *Weissella viridescens* FemX in complex with UDP-MurNAc-pentapeptide (UM5P) substrate (PDB ID: 1P4N [152]); and (**C**) *E. coli* leucyl/phenylalanyl-tRNA protein transferase (EcLFT) in complex with puromycin (PDB ID: 2DPT [161]). The conserved and non-conserved motifs of the GNAT domains are colored as in Figure 3.

Structural analysis of FemA of *Staphylococcus aureus* (SaFemA, EC 2.3.2.17) revealed that SaFemA is monomeric in the crystal and is composed of two GNAT domains (domain 1: residues 1–144 and domain 2: residues 145–395). Domain 2 contains a long coiled subdomain consisting of a pair of antiparallel α-helices (residues 246–307) inserted between the β3 and β4 strands (Figure 17A) [150]. The presence of this helical “arm” is a unique structural feature of SaFemA. Comparisons with bacterial seryl-tRNA synthetase suggested that the helical arm forms the glycyl-tRNA substrate binding site [150,157]. The two GNAT domains together form an L-shaped channel that traverses SaFemA and is thought to accommodate a binding site for a peptidoglycan precursor. This is an interesting example of an enzyme that utilizes the GNAT fold to catalyze a reaction that does not involve AcCoA.

FemX from *Weissella viridescens* (WvFemX, EC 2.3.2.10) catalyzes the transfer of l-Ala to the side-chain amino group of Lys present at the third position of the *W. viridescens* peptidoglycan precursor UDP-MurNAc-pentapeptide, using Ala-tRNA^Ala^ as a donor substrate. Structural analysis of WvFemX in the free form and in complex with the acceptor substrate (UDP-MurNAc-pentapeptide) or reaction product (UDP-MurNAc-pentapeptide-Ala) revealed that WvFemX forms a dimer in the crystal [158]. The WvFemX monomer harbors two GNAT domains: an N-terminal (residues 1–145 and 317–335) and a C-terminal (residues 146–316) one. The overall topology of WvFemX is similar to that of SaFemA, except for the helical arm that is absent in WvFemX (Figure 17B). The N-terminal GNAT domain of WvFemX interacts with UDP-MurNAc-pentapeptide, while the C-terminal GNAT domain interacts with the donor Ala-tRNA. Site-directed mutagenesis confirmed the important roles of K36, R211 and Y215 for the substrate binding [159]. The orientation of the aromatic ring and the hydroxyl group of Y254 (β10 strand) suggested its involvement in the tRNA binding [152]. K305 and F304 were shown to be essential for the catalytic activity [158]. K305 could contribute to the stabilization of the negative charge that develops on the carbonyl oxygen of l-Ala after the nucleophilic attack of the ester bond of Ala-tRNA^Ala^ by the amine group of UM5P [152]. Interestingly, in contrast to the GNAT acetyltransferases, FemX aminoacyl transferase follows a novel substrate-aided transfer mechanism rather than using a general catalytic base or acid [158].

Leucyl/phenylalanyl-tRNA protein transferase of *E. coli* (EcLFT, EC 2.3.2.6) shows significant topological and structural similarities to FemABX enzymes, although it acts on a different substrate. It catalyzes the transfer of leucine or phenylalanine (and, to a lesser extent, methionine or tryptophan) to the N-terminal Arg or Lys residue of proteins, using Leu-tRNA^Leu^ or Phe-tRNA^Phe^ as the second substrate [152,160,161]. It plays an important role in protein degradation [161]. The structural analysis of EcLFT in complex with an aminoacyl-tRNA analog puromycin showed that the EcLFT monomer has a C-terminal GNAT domain comprising residues 63 to 232 [161] (Figure 17C) that has a similar topology to the C-terminal GNAT domain of WvFemX and SaFemA. The N-terminal domain of EcLFT has a different, non-GNAT, fold. The 6-*N*,*N*-dimethyladenine moiety mimicking the 3′-terminal adenosine of aminoacyl-tRNAs is stabilized by a π–π stacking interaction with W49 from the N-terminal domain, which suggests that the N-terminal domain interacts with the acceptor substrate. The *p*-methoxybenzyl moiety of puromycin, mimicking the amino-acid moiety of Leu-tRNA^Leu^ or Phe-tRNA^Phe^, is harbored in a highly hydrophobic pocket formed by residues M144, F153, L170, F173 and I185. Mutations of the residues that form stabilizing interactions with puromycin significantly decreased the activity of EcLFT, confirming their important role in the recognition of aminoacyl-tRNAs. Analysis of the model of the EcLFT complex with tRNA and a substrate protein suggested that the side chains of E156 and Q188 serve to anchor the positively charged side chain of the N-terminal Arg or Lys of the acceptor protein close to the aminoacyl bond of the aminoacyl-tRNA to promote the formation of the peptide bond. These residues are therefore thought to define the acceptor substrate specificity of EcLFT [161].

### 2.16. Protein N-Myristoyltransferase Family (NMT, EC 2.3.1.97)

N-myristoyltransferase (NMT) facilitates the transfer of myristate (a 14-carbon saturated fatty acid) from myristoyl CoA (Myr-CoA) to the N-terminal glycine residue of various fungal, eukaryotic, protozoan and viral proteins [162,163,164,165,166,167]. Myristoylation of proteins can promote reversible protein–protein interactions, interactions between proteins and the cellular membrane, or enhance protein stability [165]. However, this modification, or its abnormal levels, was also linked to many human diseases including cancer, genetic disorders and viral infection [165].

**Figure 18:**
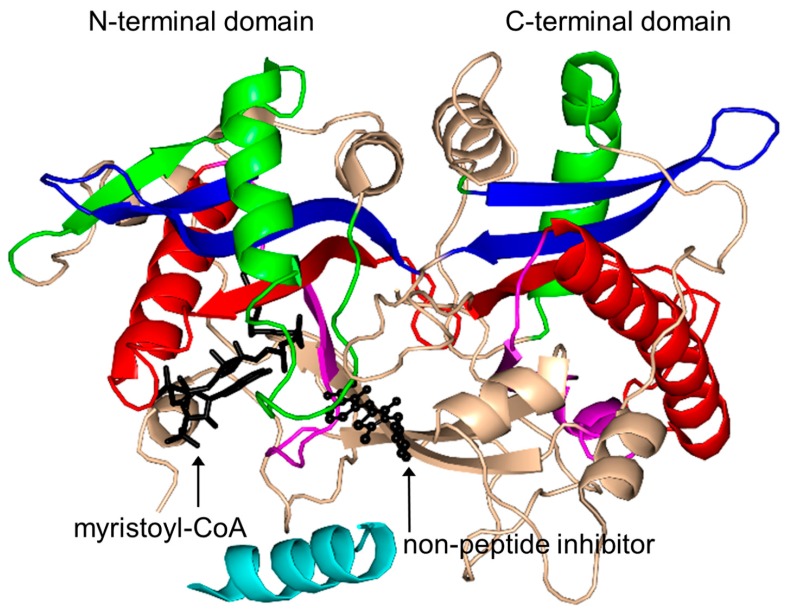
Cartoon representation of the structure of yeast *N*-myristoyltransferase in complex with myristoyl CoA and the non-peptide inhibitor (*Z*)-3-benzyl-5-(2-hydroxy-3-nitrobenzylidene)-2-thioxothiazolidin-4-one (PDB ID: 2P6F [169]). The conserved and non-conserved motifs of the GNAT domain, cofactor and inhibitor are colored as in Figure 3.

NMT (451 aa) from *Candida albicans* (CaNMT) is a monomeric enzyme that, like FemABX aminoacyl transferases, harbors two GNAT domains. It has an internal two-fold symmetry, likely as a result of gene duplication, although the two domains show very limited sequence identity [168]. Structural analysis of its homolog from *S. cerevisiae* (ScNMT) in complex with Myr-CoA and a non-peptide inhibitor [3,169] suggested that its N-terminal region (residues 4–30), colored cyan in Figure 18, plays an important role in the recognition of both Myr-CoA and the peptide substrate. The C-terminal GNAT domain accommodates the peptide substrate binding site, occupied by the inhibitor molecule in the ternary complex, whilst the N-terminal GNAT domain accommodates Myr-CoA [169]. Comparison of the structures of the NMT homologs from yeast, human, fungi and parasites (Table 1) revealed that while the Myr-CoA binding site is conserved, the peptide substrate binding site differs significantly among NMTs from different species, which reflects their different peptide substrate specificities [170]. In common with GNAT acetyltransferases, NMT follows an ordered Bi-Bi catalytic reaction mechanism [163]. The backbone amides of both F170 and L171 generate the oxyanion hole that polarizes the thioester carbonyl moiety of Myr-CoA and contributes to the stabilization of the tetrahedral intermediate generated during the transfer reaction [3]. The carboxylate moiety of the C-terminal residue L455 would be within hydrogen bonding distance from the amino group of the N-terminal glycine of the peptide substrate, indicating that L455 could serve as a general base deprotonating the substrate in the first reaction step. Mutagenesis studies confirmed the essential role of L455 in catalysis [171]. Within the GNAT superfamily, the involvement of the C-terminal carboxylate group in catalysis is unique to NMT [172]. Structures of CaNMT homologs from other sources, including *Plasmodium* [173,174,175,176,177], *Leishmania* [178,179,180,181], *Treponema* [182], fungus [183] and human [184] have also been reported in literature.

### 2.17. Mycothiol Synthase Family (MshD, EC 2.3.1.189)

Mycothiol synthase (MshD, *M*_W_ 33.6 kDa), encoded by the *Rv0819* gene of *M. tuberculosis*, catalyzes the final step in the biosynthesis of low molecular weight organosulfur compound mycothiol (MSH) from 1-l-myo-inositol-1-phosphate [185]. It acetylates the cysteinyl amino group of l-cysteine-1-d-myo-inosityl-2-amido-2-deoxy-α-d-glucopyranoside (Cys-Gln-Ins) using AcCoA as an acetyl donor to generate MSH (AcCys-Gln-Ins). MSH is the major thiol compound in *Mycobacteria* and in most actinomycetes. It plays a significant role in neutralization of electrophiles, oxidative stress, antibiotic resistance and oxidation of formaldehyde in *M. tuberculosis* [185].

**Figure 19:**
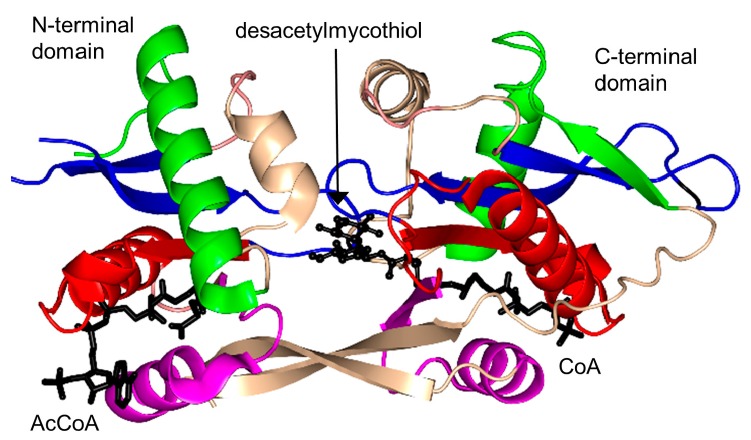
Cartoon representation of the structure of *M. tuberculosis* MshD in complex with desacetylmycothiol, AcCoA and CoA (PDB ID: 2C27 [187]). The conserved and non-conserved motifs of the GNAT domain, cofactor and substrate are colored as in Figure 3.

MshD exists as a monomer both in solution and in the crystal [186,187]. Structural analysis of MshD in complex with AcCoA revealed that this enzyme harbors two GNAT domains; residues 1–140 form the N-terminal GNAT domain while residues 151–315 form the C-terminal one (Figure 19). The pyrophosphate binding “P-loop” and the V-shaped AcCoA binding cleft are present in both domains. However, the structure suggested that only the C-terminal domain is active, because in the N-terminal GNAT domain the acetyl group of AcCoA is positioned out of reach for the acceptor substrate. Analysis of the ternary complex of MshD with CoA and the natural substrate desacetylmycothiol (DAM) showed that only one DAM molecule binds in the central cavity between the two GNAT domains [187]. This indicates that the non-catalytic N-terminal domain provides residues that form part of the substrate-binding site, and is therefore important for function. Kinetic analysis of MshD showed a bell-shaped pH dependence of *V*_max_ and *V*/*K*_DAM_, consistent with the involvement of a catalytic base with p*K*a of 6.6–7.0 and a catalytic acid with p*K* of 8.7–9.2 in the acetyl transfer reaction. The initial velocity pattern of MshD confirmed the direct transfer of the acetyl group from AcCoA to DAM via formation of a tetrahedral-intermediate. The structure showed that the primary amine moiety of DAM is positioned in an orientation that favors the direct nucleophilic attack on the acetyl group of AcCoA bound to the catalytically active C-terminal GNAT domain. Structural analysis revealed that E234 at the C-terminal domain could accept a proton from the primary amine of DAM via a well-ordered water molecule, and thus could serve as a general base. The backbone amide nitrogen of L238 forms a hydrogen bond with the carbonyl oxygen to facilitate proper positioning of the acetyl group of AcCoA and could play an important role in stabilizing the tetrahedral reaction intermediate. The hydroxyl group of Y294 is positioned near to the sulfur atom of CoA (~3.6 Å) and could act as a general acid to donate a proton to the leaving thiolate ion.

## 3. Oligomerization of GNAT Superfamily Enzymes

**Figure 20:**
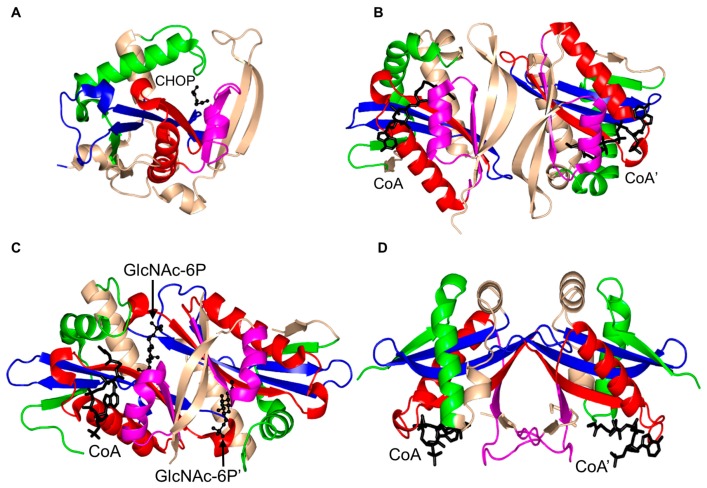

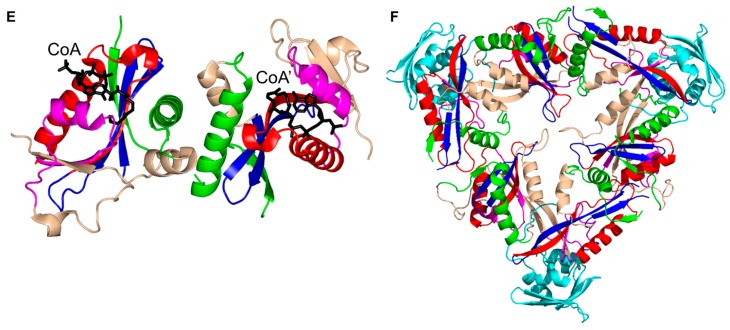
Representative oligomeric structures observed in the GNAT superfamily. (**A**) Monomeric ScMpr1; (**B**) dimeric StRimL where the β-strands from the two monomers at the dimer interface are arranged to form a continuous β-sheet; (**C**) dimeric interface where the C-terminal β-strands are interchanged between the monomers (ScGNA1); (**D**) a 12-strand β-barrel at the dimer interface of SmAAC(3)-Ia; (**E**) dimer interface involving α-helices from both monomers (hPCAF); and (**F**) one-half of the MtEis hexamer (two three-fold symmetrical trimers shown in this Figure assemble into an asymmetric “sandwich”). The conserved and non-conserved motifs of the GNAT domain, cofactors and substrates are colored as in Figure 3.

**Figure 21:**
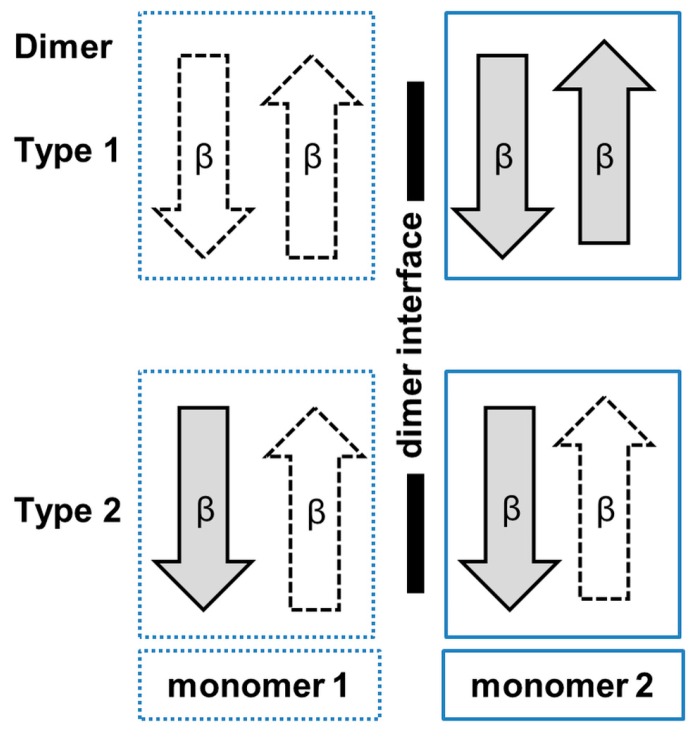
Schematic figure illustrating the topology of the β-strands found in the two most common types of dimer interface in GNAT proteins. Strands from one monomer are colored white, and those from the second monomer are colored gray.

Previous studies on GNAT superfamily members suggested that many of them are oligomeric, and that oligomerization is important for their function. Most of the GNAT transferases are dimeric (Figure 20), although examples of monomeric (Figure 20A), trimeric, tetrameric, hexameric (Figure 20F) and dodecameric enzymes have also been discussed in this review. Four different dimerization modes have been observed. One of the most frequently occurring arrangements of the β-strands at the dimer interface is where the C-terminal β-strands from each monomer come together to form a continuous β-sheet (Figure 20B and Figure 21). This mode of dimerization was observed in the crustal structures of StRimL, EfAAC(6)-Ii, HpPseH, MtRv0802c, hSSAT and EcWecD [10,107,108,113,126]. In the second common type of dimers in the GNAT superfamily, the monomers exchange their C-terminal β-strands so that they are inserted in the core β-sheet of the opposite monomer (Figure 20C and Figure 21). This type of dimerization was found in ScHpa2, SeAAC(6′)-Iy and ScGNA1 [22,76,99]. The third type of dimerization is exemplified by the crystal structure of SmAAC(3)-Ia [5]. The two SmAAC(3)-Ia monomers associate in such a way that the 12 β-strands form a β-barrel at the dimer interface (Figure 20D). The fourth type of a dimer is represented by the crystal structure of hPCAF where the dimer is stabilized via formation of a four-helical bundle at the dimer interface (Figure 20E).

## 4. General Catalytic Mechanism of GNAT Superfamily Members

**Figure 22:**
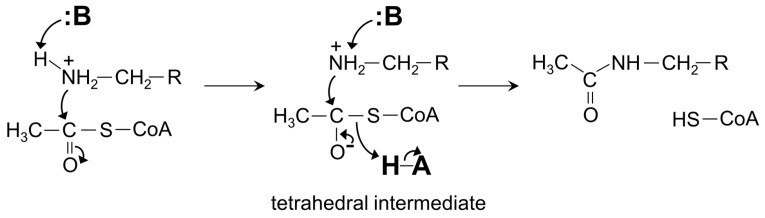
A common general direct acyltransfer mechanism of GNAT superfamily members.

Cumulatively, structural and kinetic studies on the GNAT enzymes and their complexes with substrates or inhibitors demonstrated that the members of this superfamily follow a common catalytic mechanism that involves a direct transfer of an acyl group from a donor substrate (usually AcCoA) on the amino group of an acceptor substrate. With the exception of Mpr1 that catalyzes acetylation of secondary amines, all GNAT enzymes characterized to date act on primary amino groups. Generally, the amino group of the acceptor substrate needs to be deprotonated to perform a direct nucleophilic attack on the carbonyl carbon of the enzyme-bound AcCoA. Most GNAT enzymes have an amino acid (usually Glu, Asp or Ser) near their active site that serves as a general base that extracts a proton from the amino group of the substrate [10,31,65,93]. Following the nucleophilic attack on AcCoA a transient zwitterionic tetrahedral intermediate form (Figure 22), the breakdown of which occurs through a proton transfer from a general acid (usually Tyr or Ser) and may involve deprotonation of the tetrahedral intermediate by a general base, after which the acetylated product is released [10,137]. He and colleagues [114] reported that 62% of the characterized GNAT enzymes have a conserved Tyr residue that serves as a general acid in the reaction, and 36% of them have a conserved Glu residue that serves as a general base.

In some GNAT enzymes, two or more amino acid residues together serve to abstract a proton from the amino group of the substrate. Deprotonation of the acceptor substrate by a general base may also occur via ordered water molecule(s) [23,45,80,132], although some GNAT enzymes do not utilize a specific amino acid residue for the substrate deprotonation. For example, the PA4794 protein uses well-ordered water molecules for the deprotonation step [137], while the positive charge around the entrance to the active site of ScHpa2 is thought to favor a deprotonated state of the substrate, thus negating a requirement for a specific general base residue [76]. Substrates of some GNAT enzymes (for example, *N*-acetylglucosamine-6-phosphate, the substrate for ScGNA1) have a lower p*K*_a_ than that of other GNAT acceptor substrates and bind to the enzyme in a deprotonated form, eliminating the need for a general base [99].

Analysis of the pH dependence of the enzymatic activity combined with mutagenesis and structural studies suggested that some members of the GNAT superfamily (e.g., ScGCN5 and SeAAC(6′)-Iy) catalyze the transfer reaction via water—rather than amino acid residue-mediated protonation of the tetrahedral intermediate; these enzymes appear to have only one catalytic residue (general base) [39,188]. Interestingly, in contrast to GNAT acetyltransferases, the FemX aminoacyl transferase member of this superfamily follows a substrate-aided transfer mechanism rather than using a general catalytic base or acid residing on the protein [158]. The hydrogen bond between the carbonyl group of acyl-CoA and the main-chain amide of a residue on the β4 strand is a conserved feature found in many GNAT enzymes. This bond likely stabilizes the tetrahedral reaction intermediate [3,10,23,31,68,73,108,147].

Some GNAT enzymes (for example, hSSAT [132] and (SeAAC(6′)-Iy [22]) follow a random kinetic mechanism, where either AcCoA or an acceptor substrate can bind to the free enzyme first. There are also examples of GNAT transferases that follow an ordered kinetic mechanism, where either the acceptor substrate binds first followed by AcCoA (e.g., rat liver SSAT [128]) or AcCoA binds first, followed by the substrate (e.g., EcAAC(6′)-Ib [25] and OaAANAT [77]. The structural study of OaAANAT revealed that binding of AcCoA to the enzyme induces major structural rearrangements at its active site that complete the serotonin binding pocket. This provided a structural basis for the ordered sequential mechanism in OaAANAT.

## 5. Association of Expression of GNAT Enzymes with Cancer

GNAT enzymes are known to play a role in a wide range of human diseases including cancer, diabetes and asthma. Acetylation of histone and non-histone proteins by GNATs controls gene expression, regulation of transcription factors, DNA replication, DNA repair, cell cycle progression, cell signaling pathways and metabolism (Table 2) [44,46,189,190], and misregulation of the histone *N*-acetyltransferase (HAT) activity has been linked with cancer, asthma and viral infections [9,10,189,191,192]. A recent review by Kaypee et al. [189] summarized the role of aberrant histone acetylation in tumor development and discussed altered an HAT profile as a diagnostic tool for early detection of cancer. Furthermore, it has been reported that a substantial increase in HAT1 expression occurs during progression of liver and colon cancer [44,193]. In addition, abnormal expression of PCAF and GCN5 has been linked to different types of human cancers including glioma, NSCLC (non-small cell lung cancer), HCC (hepatocellular carcinoma), and colon, lung, oral, prostate and ovarian cancer [189,194,195]. A recent study showed that attenuation of GCN5 expression inhibited the growth of colon cancer cells and promoted their apoptosis [195]. PCAF plays an important role in the regulation of activities of several oncogenes and tumor repressors and thereby has a significant effect on cancer development. PCAF can prevent the progression of HCC by inhibiting the serine/threonine protein kinase 1 [189,196]. There is growing evidence that HATs, PCAF and GCN5 can be exploited as anticancer targets. Current knowledge on inhibitors targeting GNAT enzymes for therapeutic use has been reviewed elsewhere [192].

Downregulation of expression of a GNAT enzyme from a different family—arylalkylamine *N*-acetyltransferase (AANAT)—correlated with development of the biliary cancer, cholangiocarcinoma [197]. Elevated AANAT expression has showed an inhibitory effect on the development of cholangiocarcinoma and increased apoptosis of the cholangiocarcinoma cell line in an in vitro assay. The results of that study suggested the therapeutic importance of AANAT for management of biliary cancer [197].

Altered cellular expression of the polyamine regulatory GNAT enzyme spermidine/spermine *N*^1^-acetyltransferase (SSAT) has been linked with a variety human diseases including cancer and rheumatoid arthritis (reviewed in [123,198]). A recent study reported that elevated expression of SSAT is involved in the development of prostate carcinoma and in metastasis [199]. Induction of SSAT by polyamine analogs (for example, BE-3-3-3) resulted in apoptosis of tumor cells which suggested that SSAT can be a potential target for the development of novel therapeutics [123].

Glucosamine-6-phosphate *N*-acetyltransferase (GNA1) catalyzes the synthesis of the precursor UDP-*N*-acetylglucosamine (UDP-GlcNAc) in the hexosamine biosynthetic pathway. UDP-GlcNAc serves as a donor substrate for *O*-GlcNAcylation of a wide variety of cytosolic and nuclear proteins, and thereby plays a significant role in diverse cellular functions including DNA replication, gene transcription, cell growth and metabolism. Abnormal regulation of *O*-GlcNAcylation has been linked with a number of human diseases including diabetes and breast cancer [200]. Elevated *O*-GlcNAcylation plays a crucial role in the progression of breast cancer [200,201]. Therefore, GlcNAcylation could be exploited as a novel target for the development of anticancer therapeutics.

Aberrant expression/activity of human *N*-myristoyltransferase (NMT) was observed in colon, stomach, breast, lung, gallbladder and brain cancer (reviewed in [184,202,203]. Inhibiting NMT1 activity resulted in reduced cancer progression in a murine model and increased apoptosis in different cancer cell lines [184,202]. Development of potential therapeutic anticancer drugs targeting human NMTs has been reviewed elsewhere [202].

## 6. GNAT Enzymes as Potential Targets for Antimicrobial Agents

Structural studies on the complexes of microbial GNAT enzymes with their substrates or substrate analogs have provided useful information for the structure-guided design of compounds that can be potentially developed into novel antimicrobial agents. Inhibition of bacterial aminoglycoside *N*-acetyltransferases (AACs) has been studied extensively because these enzymes modify aminoglycosides that are widely used in clinical settings, thereby conferring bacterial resistance. It was shown that a bisubstrate analog consisting of aminoglycoside covalently linked with CoA inhibited *E. coli* AAC in an in vitro assay [27]. However, that inhibitor was ineffective in an in vivo assay, likely due to inability to penetrate the bacterial cell wall. Subsequently, it was shown that truncated aminoglycoside-CoA analogs inhibited SeAAC(6)-Iy at micromolar concentrations in vitro [22], and EfAAC(6)-Ii—at nanomolar concentrations, both in vitro and in vivo [36,37,38], demonstrating that they have potential as lead compounds in the development of novel therapeutics effective against aminoglycoside-resistant bacterial strains expressing AAC(6′).

Lombes and co-workers [204] used NMR-based approaches to identify non-aminoglycoside-like fragments that showed micromolar-range inhibition of a different type of AAC(6′) of *E. coli* (EcAAC(6)-Ib). Recently, an EcAAC(6′)-Ib inhibitor (1-[3-(2-aminoethyl)benzyl]-3-piperidin-1-ylmethyl)pyrrolidin-3-ol), identified by an in silico docking approach [32], was shown to be effective in preventing the growth of an amikacin-resistant *A. baumannii* clinical strain that expresses AAC(6′)-Ib [32].

A different GNAT family that has been targeted in efforts to identify novel antimicrobial compounds is protein *N*-myristoyltransferases (NMTs). Fungal and parasitic NMTs are essential enzymes that have different substrate specificities compared to their human homologs. In the case of *Candida albicans*, an opportunistic human fungal pathogen that can cause systemic infections in immunosuppressed patients, benzofuran inhibitors of *C. albicans* NMT showed fungicidal activity in vivo [205]. Pharmacokinetic study and analysis of structure–activity relationships demonstrated that these inhibitors have high selectivity over human NMT and are therefore good candidates for development into antifungal drugs for human use [205].

Recently, Rackham and colleagues [174,175] identified benzothiophene ring-containing NMT inhibitors that target both blood and liver stage forms of the malaria parasite *Plasmodium falciparum*. Furthermore, a pyrazole sulfonamides inhibitor of parasitic NMT that shows antimicrobial activity in the initial hemolymphatic stage of human African sleeping sickness caused by *Trypanosoma brucei*, was identified [181]. Optimization of this compound led to the discovery of *T. brucei* NMT inhibitors with higher blood-brain barrier permeability and improved selectivity over human NMT, that showed partial efficacy in stage 2 (when the parasite invades the central nervous system) in the mouse model of trypanosomiasis [206]. In addition, *T. cruzi* NMT was recently validated as a potential target for the development of drugs that target the mammal-dwelling stages of Chagaz disease [207]. It is anticipated that the knowledge of the structural basis behind the reaction and substrate specificity of the enzymes from the GNAT superfamily, gained through the studies of their complexes with inhibitors and substrates, can be exploited for the development of novel therapeutics to combat emerging multidrug-resistant microbial infections.

## 7. Conclusions

The last two decades have seen a dramatic increase in the amount of structural information available on GNAT enzymes. Although overall amino acid sequence identity among the members of this superfamily is very low, most share common core structural features. GNAT enzymes show significant diversity in their substrate specificities and play an important role in many biological processes. It is anticipated that the knowledge of the structural basis behind the reaction and substrate specificity of these enzymes, gained through studies of their complexes with inhibitors and substrates, can be exploited for the development of novel therapeutics to combat emerging multidrug-resistant microbial infections and other life-threatening human diseases including cancer and metabolic disorders.

Future Issues:

What roles (allosteric, cooperative, etc.) do different types of oligomerization play in the catalytic activity and specificity of GNAT enzymes?

Can structures of GNAT enzymes of unknown function determined by the Structural Biology Consortiums in combination with high-throughput ligand screening be used to predict their putative substrates?

How does the three-dimensional structure of a GNAT enzyme define whether it follows a random or an ordered kinetic mechanism?

How do GNAT enzymes recognize their partner proteins within the same pathway?

Does posttranslational modification of GNAT enzymes occur and how does it affect their structure and function?

## Figures and Tables

**Table 1:** General control non-repressible 5-related *N*-acetyltransferase (GCN5) superfamily members of a known structure (continues on next page).

Family	Name	EC No.	Source	Substrates	PBD IDs	References
Aminoglycoside *N*-acetyltransferases	MtAAC(2′)-Ic	2.3.1.-	*M. tuberculosis*	aminoglycosides	1M44/1M4D/1M4G/1M4I	[23]
	SmAAC(3)-Ia	2.3.1.81	*S. marcescens*	aminoglycosides	1BO4	[5]
	EcAAC(6′)-Ib	2.3.1.82	*E. coli*	aminoglycosides	1V0C/2BUE/2VQY	[25]
	SeAAC(6′)-Ib_11_	2.3.1.82	*S. enterica*	aminoglycosides	2PR8/2PRB/2QIR	[30]
	SwAAC(6′)-Ie	2.3.1.-	*S. warneri*	aminoglycosides	4QC6	[208]
	EfAAC(6′)-Ii	2.3.1.82	*E. faecium*	aminoglycosides	1B87/1N71/2A4N	[13,21,34]
	SeAAC(6′)-Iy	2.3.1.82	*S. enterica*	aminoglycosides	1S60/1S3Z/1S5K/2VBQ	[22,39]
	MtEis	2.3.1.-	*M. tuberculosis*	aminoglycosides	3R1K/3RYO/3SXN/3UY5/4JD6	[19,41,42]
Histone *N*-acetyltransferases	hHAT1	2.3.1.48	*H. sapiens*	histone H4	2P0W	[45]
	ScHAT1	2.3.1.48	*S. cerevisiae*	histone H4	1BOB	[6]
	ScEsa1	2.3.1.48	*S. cerevisiae*	histone H4	1MJ9/1MJA/1MJB	[57,58]
	ScGCN5	2.3.1.48	*S. cerevisiae*	histone H3, H4	1YGH	[65]
	TtGCN5	2.3.1.48	*T. thermophila*	histone H3	1QSN/1QSR/1QST/5GCN/1M1D/1PU9/1PUA/1Q2C	[67,68,69,70]
	hGCN5	2.3.1.48	*H. sapiens*	histone H3, H4, H2b	1Z4R	[66]
	hPCAF	2.3.1.48	*H. sapiens*	histone H3, H4	1CM0/4NSQ	[72,73]
	ScHpa2	2.3.1.48	*S. cerevisiae*	histone H3, H4	1QSM/1QSO	[76]
Non-histone protein *N*-acetyltransferases	αTAT1	2.3.1.108	*H. sapiens*	α-tubulin	4GS4	[79]
	MtPAT	3.4.1.-	*M. tuberculosis*	-	4AVA /4AVB/4AVC	[81]
	SsPAT	2.3.1-	*S. solfataricus*	-	3F8K	[78]
	hNaa50p	2.3.1.-	*H. sapiens*	peptides	3TFY	[80]
Arylalkylamine *N*-acetyltransferases	OaAANAT	2.3.1.87	*O. aries*	2-arylethylamines	1B6B/1CJW/1KUV/1KUX/1KUY/1L0C	[77,86,209,210]
	hAANAT	2.3.1.87	*H. sapiens*	2-arylethylamines	1IB1	[211]
	DmAANAT	2.3.1.87	*D. melanogaster*	2-arylethylamines	3TE4	[93]
	AaAANAT	2.3.1.87	*A. aegypti*	histamine, arylalkylamines, hydrazine	4FD5/4FD4,4FD6/4FD7	[94]
Glucosamine-6-phosphate *N*-acetyltransferases	ScGNA1	2.3.1.4	*S. cerevisiae*	d-glucosamine 6-phosphate	1I1D/1I12/1I21	[99]
	AfGNA1	2.3.1.4	*A. fumigatus*	d-glucosamine 6-phosphate	2VEZ/2VXK	[100,212]
	hGNA1	2.3.1.4	*H. sapiens*	d-glucosamine 6-phosphate	3CXP/3CXQ/3CXS	[101]
	TbGNA1	2.3.1.4	*T. brucei*	d-glucosamine 6-phosphate	3I3G	[213]
	AtGNA1	2.3.1.4	*A. thaliana*	d-glucosamine 6-phosphate	3T90	[102]
	CeGNA1	2.3.1.4	*C. elegans*	d-glucosamine 6-phosphate	4AG7/4AG9	[103]
MccE	EcMccE	-	*E. coli*	aspartyl-tRNA synthetase	3R95/3R96/3R9E/3R9F	[23]
Pseudaminic acid biosynthesis protein H	HpPseH	2.3.1.202	*H. pylori*	UDP—linked sugar	4RI1	[107]
	CjPseH	2.3.1.202	*C. jejuni*	UDP—linked sugar	4XPK/4XPL	[109]
WecD	EcWecD	2.3.1.210	*E. coli*	dTDP-4-amino-4,6-dideoxy-α-d-galactose	2FS5/2FT0	[113]
Tabtoxin resistance protein	PsTTR	2.3.1.-	*P. syringae*	tabtoxin	1GHE	[114]
Mpr1	ScMpr1	3.4.1.-	*S. cerevisiae*	l-azetidine-2-carboxylic acid	3W6S/3W6X/3W91	[119]
Spermidine/spermine *N*^1^-acetyltransferases	BsPaiA	2.3.1.57	*B. subtilis*	spermidine/spermine	1TIQ	[126]
	TaPaiA	2.3.1.57	*T. acidophilum*	spermidine/spermine	3FIX/3FIX/3NE7/3NE7	[130]
	hSSAT	2.3.1.57	*H. sapiens*	spermidine/spermine	2BEI/2B3U/2B3V/2B58/2B4B/2B4D/2B5G/2F5I/2G3T/2JEV	[129,132,133,214]
	MmSSAT	2.3.1.57	*M. musculus*	spermidine/spermine	3BJ7/3BJ8	[128]
	VcSpeG	2.3.1.57	*V. cholerae*	spermidine/spermine	4NCZ/4JJX/4MHD/4MI4/4R57/4R87	[127]
C-terminal *N*^ε^-lysine protein acetyltransferases	PA4794	2.3.1.-	*P. aeruginosa*	C-terminal lysine containing peptide	3PGP/4KOS/4KOT/4KOU/4KOV/4KOW/4KOX/4KOY/4KUA/4L89	[167]
	MtRv1347c	2.8.3.-	*M. tuberculosis*	-	1YK3	[138]
Ribosomal protein *N*^α^-acetyltransferases	SeRimI	2.3.1.128	*S. enterica*	ribosomal protein S18	2CNS/2CNT	[147]
	StRimL	2.3.1.-	*S. typhimurium.*	ribosomal protein L7/L12	1S7F/1S7K/1S7L/1S7N	[108]
	BsYadF	-	*B. subtilis*	ribosomal protein L12	1NSL	[149]
Succinyltransferase	MtRv0802c	2.8.3.-	*M. tuberculosis*	-	2VZY/2VZZ	[4]
FemABX aminoacyl transferases	SaFemA	2.3.2.17	*S. aureus*	peptidoglycan precursor, peptides	1LRZ	[150]
	WvFemX	2.3.2.10	*W. viridescens*	peptidoglycan precursor, peptides	1P4N/1NE9/1XE4/1XF8/1XIX/4II9	[152,158,159]
	EcLFT	2.3.2.6	*E. coli*	N-terminal Arg/Lys containing proteins	2DPS/2DPT	[161]
Protein *N*-myristoyltransferases	CaNMT	2.3.1.97	*C. albicans*	N-terminal glycyl-peptides	1NMT/1IYK/1IYL	[168,215]
	LdNMT	2.3.1.97	*L. donovani*	N-terminal glycyl-peptides	2WUU	[216]
	LmNMT	2.3.1.97	*L. major*	N-terminal glycyl-peptides	2WSA/3H5Z/4A2Z/4A30/4A31/4A32/4A33/4CGL/4CGM/4CGN/4CGO/4CGP/4C68/4C7H/4C7I/4CYN/4CYO/4CYP/4CYQ	[178,179,180,181]
	ScNMT	2.3.1.97	*S. cerevisiae*	N-terminal glycyl-peptides	2NMT/1IIC/1IID/2P6E/2P6F/2P6G	[3,169,172]
	PvNMT	2.3.1.97	*P. vivax*	N-terminal glycyl-peptides	4B10/4B11/4B12/4B13/4B14/4A95/4BBH/2YNC/2YND/2YNE/4CAE/4CAF	[173,174,175,176,177]
	TbNMT	2.3.1.97	*T. brucei*	N-terminal glycyl-peptides	2WSA/3H5Z/4A2Z	[182]
	AfNMT	2.3.1.97	*A. fumigatus*	N-terminal glycyl-peptides	4CAX/4CAV/4CAW	[183]
	hNMT	2.3.1.97	*H. sapiens*	N-terminal glycyl-peptides	4C2X/4C2Y/4C2Z	[184]
Mycothiol synthase	MtMshD	2.3.1.189	*M. tuberculosis*	des-acetylmycothiol	1OZP/1P0H/2C27	[186,187]

**Table 2:** Functions of GNAT superfamily members.

Family	Functions	References
Aminoglycoside *N*-acetyltransferases	resistance to aminoglycoside antibiotics	[10,19,41,42]
Histone *N*-acetyltransferases	histone deposition, transcription activation, chromatin assembly, DNA repair, promoting cancer cell growth and apoptosis, amino acid biosynthesis in yeast	[43,44,46,54,55,56,63]
Non-histone protein *N*-acetyltransferases	chromatin regulation, maintaining genome integrity, mRNA and protein stability	[78,82,83,90]
Arylalkylamine *N*-acetyltransferases	xenobiotic and folate metabolism, biosynthesis of melatonin, sclerotization and neurotransmitter inactivation	[91,92,95]
Glucosamine-6-phosphate *N*-acetyltransferases	biosynthesis of peptidoglycan, chitin, hexosamine and glycophosphatidylinositol; amino sugar and nucleotide sugar metabolism	[10,11,96,97,98]
MccE	inactivation of antibiotic microcin C7	[104,105]
Pseudaminic acid biosynthesis protein H	flagellin glycosylation	[106]
WecD	biosynthesis of enterobacterial common antigen and flagella	[110,111,112]
Tabtoxin resistance protein	inactivation of tabtoxin and prevention of wildfire disease	[117,118]
Mpr1	l-proline and l-arginine metabolism, oxidative stresses resistance and freeze tolerance	[119,120,121,122]
Spermidine/spermine *N*^1^-acetyltransferases	regulation of cellular polyamine levels and polyamine metabolism	[123,124,128]
C-terminal *N*^ε^-lysine protein acetyltransferases	regulation of gene expression	[135,136]
Ribosomal protein *N*^α^-acetyltransferases	regulation of proteins function and stability, inactivation of antibiotic microcin C7	[145,146,147]
Succinyltransferase	unknown	[4]
FemABX aminoacyl transferases	peptidoglycan biosynthesis and methicillin resistance	[151,153,154]
Protein *N*-myristoyltransferases	regulation of protein–protein and protein-cellular membrane interactions, enhancement of protein stability	[165,176]
Mycothiol synthase	biosynthesis of organosulfur compounds, neutralization of electrophiles, regulation of oxidative stress, antibiotic resistance and oxidation of formaldehyde	[10,185]
